# White matter functional connectome gradient dysfunction in major depressive disorder

**DOI:** 10.1093/psyrad/kkaf008

**Published:** 2025-04-28

**Authors:** Baoxin Yu, Xiaoyi Sun, Mingrui Xia

**Affiliations:** State Key Laboratory of Cognitive Neuroscience and Learning, Beijing Normal University, Beijing 100875, China; Beijing Key Laboratory of Brain Imaging and Connectomics, Beijing Normal University, Beijing 100875, China; IDG/McGovern Institute for Brain Research, Beijing Normal University, Beijing 100875, China; State Key Laboratory of Cognitive Neuroscience and Learning, Beijing Normal University, Beijing 100875, China; Beijing Key Laboratory of Brain Imaging and Connectomics, Beijing Normal University, Beijing 100875, China; IDG/McGovern Institute for Brain Research, Beijing Normal University, Beijing 100875, China; School of Systems Science, Beijing Normal University, Beijing 100875, China; State Key Laboratory of Cognitive Neuroscience and Learning, Beijing Normal University, Beijing 100875, China; Beijing Key Laboratory of Brain Imaging and Connectomics, Beijing Normal University, Beijing 100875, China; IDG/McGovern Institute for Brain Research, Beijing Normal University, Beijing 100875, China

**Keywords:** depression, fMRI, functional connectivity, brain network, hierarchy, white matter functional network

## Abstract

**Background:**

Major depressive disorder (MDD) is a prevalent psychiatric disorder with disruptions in brain white matter (WM). While much research has focused on WM structure, the dysfunctional organization of WM in MDD remains poorly understood.

**Methods:**

Using resting-state functional magnetic resonance imaging data from 48 MDD patients and 68 healthy controls (HC), we characterized the WM functional connectome gradients across participants and identified both global and regional alterations in MDD. Furthermore, we examined the relationship between gradient properties and depressive symptom severity. External validation and sensitivity analyses were finally conducted to ensure the reliability of results.

**Results:**

The principal WM connectome gradient extended from the forceps major and superior longitudinal fasciculus to the uncinate fasciculus (UF) and anterior thalamic radiation (ATR), exhibiting a superficial-to-deep pattern in both groups. Compared to HC, MDD patients displayed a narrower gradient range and lower spatial variation, indicating a contracted WM hierarchy. At the tract-specific level, MDD patients exhibited lower gradient scores in the forceps minor, left ATR and UF, and bilateral cingulate gyrus and cingulum hippocampus, but higher gradient scores in the forceps major, bilateral inferior longitudinal fasciculus and superior longitudinal fasciculus. WM tract gradient patterns explained 37.2% of the variance in clinical severity, with the strongest contributions from the inferior fronto-occipital fasciculus, cingulum hippocampus, ATR, UF, and corticospinal tract.

**Conclusions:**

These findings highlight altered WM functional connectome gradient in MDD and their association with clinical severity, offering novel insights into the neurobiological mechanisms of the disorder and potential biomarkers for symptom evaluation.

## Introduction

Major depressive disorder (MDD) is a highly prevalent and debilitating psychiatric condition, characterized by emotional, cognitive, and somatic symptoms (James *et al*., 2018; Mathers and Loncar, 2006; World Health Organization, 2017). Diffusion tensor imaging (DTI) studies have revealed that MDD is closely associated with widespread disruptions in brain white matter (WM), manifesting both in regional tract-specific diffusion properties—indicative of underlying microstructural fiber abnormalities—and in the altered topological organization of WM structural networks (Aronica *et al*., 2022; Chen *et al*., 2021; Guo *et al*., 2024; Radoeva *et al*., 2023). For example, reductions in fractional anisotropy (FA) and increases in radial diffusivity (RD), indicative of microstructural compromise in WM tracts, have consistently been reported in key WM fiber tracts, such as the corpus callosum (CC), anterior thalamic radiation (ATR), and superior longitudinal fasciculus (SLF) (Flinkenflugel *et al*., 2024; Ji *et al*., 2023; van Velzen *et al*., 2020). These tracts play a critical role in interhemispheric communication, thalamocortical integration, and frontoparietal connectivity, all of which are vital for normal cognitive and emotional functions. Moreover, WM fibers provide the structural foundation for the macroscale human brain connectome, linking different brain regions into functional and structural networks. Localized disruptions in these tracts contribute to alterations in the topological organization of the WM network in MDD. Studies utilizing graph theoretical analysis have reported reduced global network strength and increased path length in the WM networks of patients with MDD (Repple *et al*., 2023, 2020), suggesting decreased network efficiency in information integration and transmission. These disruptions are particularly prominent in major fiber bundles connecting higher-order functional networks such as the frontoparietal network and default mode networks, and subcortical networks (Gong and He, 2015; Korgaonkar *et al*., 2014; Qin *et al*., 2014). These networks are heavily implicated in emotional regulation, mood processing, and executive functions, which are areas frequently impaired in MDD. Together, these findings highlight the critical role of WM structural abnormalities in the pathology of MDD.

While the traditional view has long considered WM tracts as solely structural components of the brain networks, emerging evidence suggests that WM also exhibits functional activity measurable through blood oxygenation level-dependent (BOLD) signals derived from functional magentic resonance imaging (fMRI) data. This functional activity in WM has often been overlooked but has gained increasing attention in recent years. Pioneering studies have demonstrated that WM exhibits organized functional connectivity during both resting-state and task-induced conditions (Ding *et al*., 2018; Gawryluk *et al*., 2014; Ji *et al*., 2017). WM functional activity is believed to reflect how neural information is transmitted across distant brain regions, facilitating inter-regional communication essential for cognitive functions. Recent fMRI studies have shown that intrinsic BOLD signal fluctuations in WM voxels can form organized functional networks, with distinct patterns of activity across different WM tracts. These tracts are often categorized into superficial, middle, and deep layers based on their BOLD activity characteristics (Huang *et al*., 2020; Li *et al*., 2019; Peer *et al*., 2017). Using gradient decomposition frameworks, researchers have identified several key WM functional gradients (Zhu *et al*., 2023). Some of these gradients parallel those observed in gray matter (GM), such as unimodal-to-transmodal and sensorimotor-to-visual gradients, suggesting a similar organizational principle that encodes and integrates sensory and cognitive information. More notably, a unique superficial-to-deep gradient has been identified, extending from the superior frontal WM to the CC. This gradient reflects the functional differentiation of fiber tracts across layers, with deep WM exhibiting higher FA and myelin water fraction compared to superficial WM, indicating an anatomical hierarchy underlying this organization (Zhu *et al*., 2023). Such findings underscore the complex functional architecture of WM and its potential role in brain network integration.

In MDD, our previous research revealed a contracted unimodal-to-transmodal gradient within GM functional networks, with gradient alterations associated with gene expression profiles and clinical outcomes (Xia *et al*., 2022). Concurrently, several fMRI studies have reported regional disruptions in WM functional activity and connectivity, particularly in superficial and deep fiber tracts (Huang *et al*., 2024; Lu *et al*., 2021; Zhao *et al*., 2021). For instance, decreased fractional amplitude of low-frequency fluctuations in the body of the CC, reduced regional homogeneity in the SLF, and diminished functional connectivity strength in the cingulate gyrus (CG) and genu of the CC have been observed (Huang *et al*., 2024; Zhang *et al*., 2021). However, the broader organization of the WM functional network in MDD, especially whether the superficial-to-deep functional gradient is disrupted, remains largely unexplored. Investigating WM functional gradient in MDD, alongside the known alterations in GM, could provide a more comprehensive understanding of the neurobiological mechanisms of MDD. Such insights may pave the way for the development of targeted interventions aimed at restoring disrupted brain network architecture in MDD.

To address this critical gap, the present study utilized a resting-state fMRI (r-fMRI) dataset comprising 116 individuals to investigate the functional connectome organization of WM in MDD. By leveraging a gradient decomposition framework, we systematically decomposed the WM functional network of each participant into distinct gradient components. This approach allowed us to examine both global and tract-specific alterations in WM connectome gradients in patients with MDD. Furthermore, we explored the relationships between these functional gradients and clinical severity, assessing whether gradient disruptions align with symptom profiles. Finally, to ensure the robustness of our findings, we conducted external validation in an independent cohort and performed sensitivity analyses across different analytical strategies, reinforcing the reliability of our results. This study aims to provide novel insights into how WM functional networks are reorganized in MDD, with a focus on understanding the hierarchical and spatial principles of functional gradient disruption.

## Methods

### Participants

The dataset was obtained from the publicly available Japanese Strategic Research Program for the Promotion of Brain Science (SRPBS) project (Tanaka *et al*., 2021) and initially included 71 patients with MDD and 124 healthy controls (HC), all recruited through the Center of Innovation at Hiroshima University. Diagnoses of MDD were conducted by expert clinicians in accordance with the Diagnostic and Statistical Manual of Mental Disorders (DSM-IV-TR or DSM-5). To confirm these diagnoses, the Mini-International Neuropsychiatric Interview (MINI) (Sheehan *et al*., 1998) was administered at the time of study participation. The severity of clinical symptoms in patients was evaluated using the Beck Depression Inventory (BDI-II). Exclusion criteria for all participants included contraindications for MRI, a history of substance or alcohol misuse, significant medical disorders, head trauma with loss of consciousness, or any neurological disorders. Written informed consent was obtained from all participants, and the data collection procedures were approved by the institutional review boards.

### Image acquisition

All participants were scanned using a Siemens Verio 3.0T scanner with a 12-channel head coil. r-fMRI data were acquired using an echo-planar imaging sequence with the following parameters: repetition time (TR) = 2500 ms, echo time (TE) = 30 ms, flip angle (FA) = 80°, matrix = 64 × 64, thickness = 3.2 mm, gap = 0.8 mm, slices = 40. The total scan duration was 10 min, yielding 244 volumes (including four dummy volumes). Participants were instructed to remain awake, minimize movement, and maintain visual fixation. Additional imaging details can be found in a previous study (Tanaka *et al*., 2021).

### Image preprocessing

The preprocessing of r-fMRI data was conducted using SPM12 (www.fil.ion.ucl.ac.uk/spm/) and the Data Processing Assistant for Resting-State fMRI (http://rfmri.org/DPARSF). To ensure signal stabilization and participant adaptation to the scanning environment, the first five time points were discarded. Subsequent preprocessing steps included slice-timing correction and realignment. Structural images were then coregistered with the preprocessed functional images and segmented into GM, WM, and cerebrospinal fluid (CSF) by Diffeomorphic Anatomical Registrations through Exponentiated Lie Algebra (DARTEL). Based on the transformation matrix produced by DARTEL, a CSF mask in Montreal Neurological Institute (MNI) space (70% threshold on SPM12 probability map) was transformed into individual functional space. The mean signal within the CSF mask and the 24 head motion parameters were regressed out from the functional images in each subject's individual space. To minimize GM interference with WM, all subsequent processing of the functional images was restricted exclusively to WM. The WM images were normalized into MNI space via structural segmentation, resampled to 3-mm isotropic voxels, and smoothed with a 6-mm full-width at half-maximum Gaussian kernel. Temporal bandpass filtering (0.01–0.08 Hz) was subsequently applied. A “scrubbing” procedure was implemented to address outlier volumes caused by excessive head motion (Power *et al*., 2012). Volumes with framewise displacement (FD) exceeding 0.5 mm, as well as two forward adjacent volumes and one backward adjacent volume, were replaced with linearly interpolated data. Data of 23 patients and 45 HCs were excluded due to maximum translation greater than 3 mm, maximum rotation greater than 3°, mean FD exceeding 0.3 mm, or more than 50% scrubbed volumes. Additionally, data of 11 HCs were further excluded due to older age, resulting in a final sample comprising 48 patients and 68 HCs.

### WM connectome gradient analysis

For each participant, a voxelwise WM functional network was constructed, and the diffusion map embedding approach (Hong *et al*., 2019; Margulies *et al*., 2016) was applied to compute the functional connectome gradient. Briefly, to reduce the computational complexity, the preprocessed r-fMRI images were first resampled to a 4-mm isotropic resolution. WM voxels were selected using the JHU DTI-based white-matter atlases with a maximum probability threshold of 0.25 (https://neurovault.org/collections/264/). A WM functional connectivity matrix was estimated by calculating Pearson's correlation coefficients between the time series of each pair of WM voxels. To focus on the most representative connections, the top 10% of connections for each voxel were retained, excluding weak connections likely to result from noise. Using these sparse connectivity profiles, cosine similarity was calculated between each pair of voxels. To address the issue of negative values in the similarity matrix, which could produce imaginary numbers in subsequent dimensionality reduction and lack clear biological meaning, the similarity matrix was further scaled into a normalized angle matrix (Larivière *et al*., 2020; Paquola *et al*., 2019). Diffusion map embedding, a nonlinear dimensionality reduction technique, was then employed to derive gradient components that explain variance in the functional connectome. This approach identifies low-dimensional embeddings within high-dimensional data (i.e. the connectivity similarity matrix in this study) and translates the relationships among connectivity profiles into distances in the high-dimensional embedding space. Voxels with similar connectivity profiles are positioned closer in this space, while nodes with dissimilar profiles are placed farther apart. Unlike linear dimensionality reduction methods (e.g. principal component analysis), diffusion map embedding constrains distances along the graph's neighborhood geometry in high-dimensional spaces, providing a stable representation of connectivity profiles. The resultant components (i.e. gradient scores) represent the projected positions of voxels along embedding axes that encode dominant differences in connectivity patterns. Gradient maps were aligned across participants using iterative Procrustes rotation to ensure comparability (Hong *et al*., 2019).

For each gradient map, several metrics were calculated to characterize its properties: the explanation ratio, gradient range, and gradient variance. The explanation ratio represents the percentage of connectivity variance accounted for by a given gradient. A higher explanation ratio indicates that the embedding axis of the gradient captures a more dominant organization of the functional network. The gradient range reflects the difference between the maximum positive and minimum negative values of the gradient scores across brain voxels. A larger range signifies greater differentiation in the encoded connectivity patterns between regions at opposite ends of the gradient. Gradient variance, represented as the standard deviation of gradient scores across the whole brain, measures heterogeneity in the connectivity structure across regions. A greater variance indicates higher connectivity diversity. Although these metrics are mathematically correlated (e.g. larger eigenvalues of a connectome gradient often result in larger ranges and variances in the corresponding eigenvectors), they provide distinct topographical insights into the gradient's characteristics. To further explore the spatial distribution of gradients, the gradient maps were segmented into 20 WM tracts based on the JHU DTI-based white-matter atlases (Table S1 and Fig. S1). The gradient scores for each tract were calculated as the average gradient score of all voxels within the tract, enabling a tract-specific analysis of connectivity patterns.

### Statistical analysis

To evaluate WM functional gradient alterations in patients with MDD, general linear models were used to compare global gradient metrics and tract-specific gradient scores between patients and controls. Age, sex, and mean FD were included as covariates. Multiple comparisons were corrected using the false discovery rate (FDR) method, with a significance threshold of *q *< 0.05.

To examine the relationships between WM functional gradients and clinical symptom severity, Pearson's correlations were first calculated between each global gradient metric and BDI-II scores in patients with MDD. Partial least squares (PLS) regression was then applied to investigate multivariable associations between tract-specific gradient scores and BDI-II scores. In this analysis, tract-specific gradient scores of the principal gradient were set as predictor variables, while BDI-II scores served as the response variable. PLS regressions identify components that are linear combinations of the predictor variables, explaining the maximum variance in the response variables. The statistical significance of the variance explained by each PLS component was evaluated using 10 000 permutation tests, where the correspondence between predictor and response variables was randomly shuffled. Similarly, the significance of the correlations between PLS scores and BDI-II scores was also assessed with 10 000 permutation tests.

### Validation analysis

(i) Effect of head motion: to further evaluate the potential residual effects of head motion on gradient metrics, we conducted two additional validation analyses. First, we calculated Pearson's correlations between mean FD and global gradient metrics (i.e. explanation ratio, gradient range, and gradient variance) across all participants. Second, a stricter mean FD threshold of 0.2 mm was applied to construct a highly motion-controlled subsample, followed by a reanalysis of WM functional gradients and gradient alterations in MDD. (ii) Different sparsity threshold for retaining functional connections: to evaluate the reliability of our results, we computed WM functional gradients using sparsity thresholds of 5% and 15%, in addition to the original 10%. The consistency of the resulting gradient maps and main findings was assessed for each participant, while the consistency of global gradient metrics and main findings was evaluated across participants, both using Pearson's correlation. (iii) External validation across cohorts: to evaluate the generalizability of our findings, we conducted an external validation in an independent cohort. Specifically, we utilized a dataset comprising 29 MDD patients and 27 HCs (Table S2) from the Center of Hiroshima Kajikawa Hospital (HKH), obtained from the publicly available Japanese SRPBS project. Using the same approach as in our main analyses, we identified the WM functional gradients and examined gradient alterations in MDD.

## Results

### Demographic and clinical characteristics

There were no significant differences in age, sex, mean FD, maximum translation, or maximum rotation between patients with MDD and HC (*P *> 0.05, Table 1, Fig. S2). However, patients with MDD exhibited significantly higher BDI-II scores compared to the HC group (*P* < 0.001, Table 1).

**Table 1:** Demographic and clinical characteristics of participants.

	MDD (*n* = 48)	HC (*n* = 68)	*t* or χ^2^/*P*
Age, mean (SD), years	44.06 (12.59)	45.96 (11.00)	−0.86/0.39
Sex (M/F)	23/25	23/45	2.34/0.13
BDI-II, mean (SD)	24.77 (8.62)	9.06 (6.39)	11.28/<0.001
Mean FD, mean (SD), mm	0.18 (0.05)	0.20 (0.06)	−1.88/0.06
Maximum translation, mean (SD), mm	1.28 (0.68)	1.24 (0.63)	0.63/0.72
Maximum rotation, mean (SD), degree	0.98 (0.50)	0.97 (0.55)	0.13/0.90

Abbreviations: MDD, major depressive disorder; HC, healthy controls; SD, standard deviation; M, male; F, female; BDI-II, Beck Depression Inventory-Second Edition; FD, framewise displacement.

### WM connectome gradient maps in patients with MDD and controls

**Figure 1:**
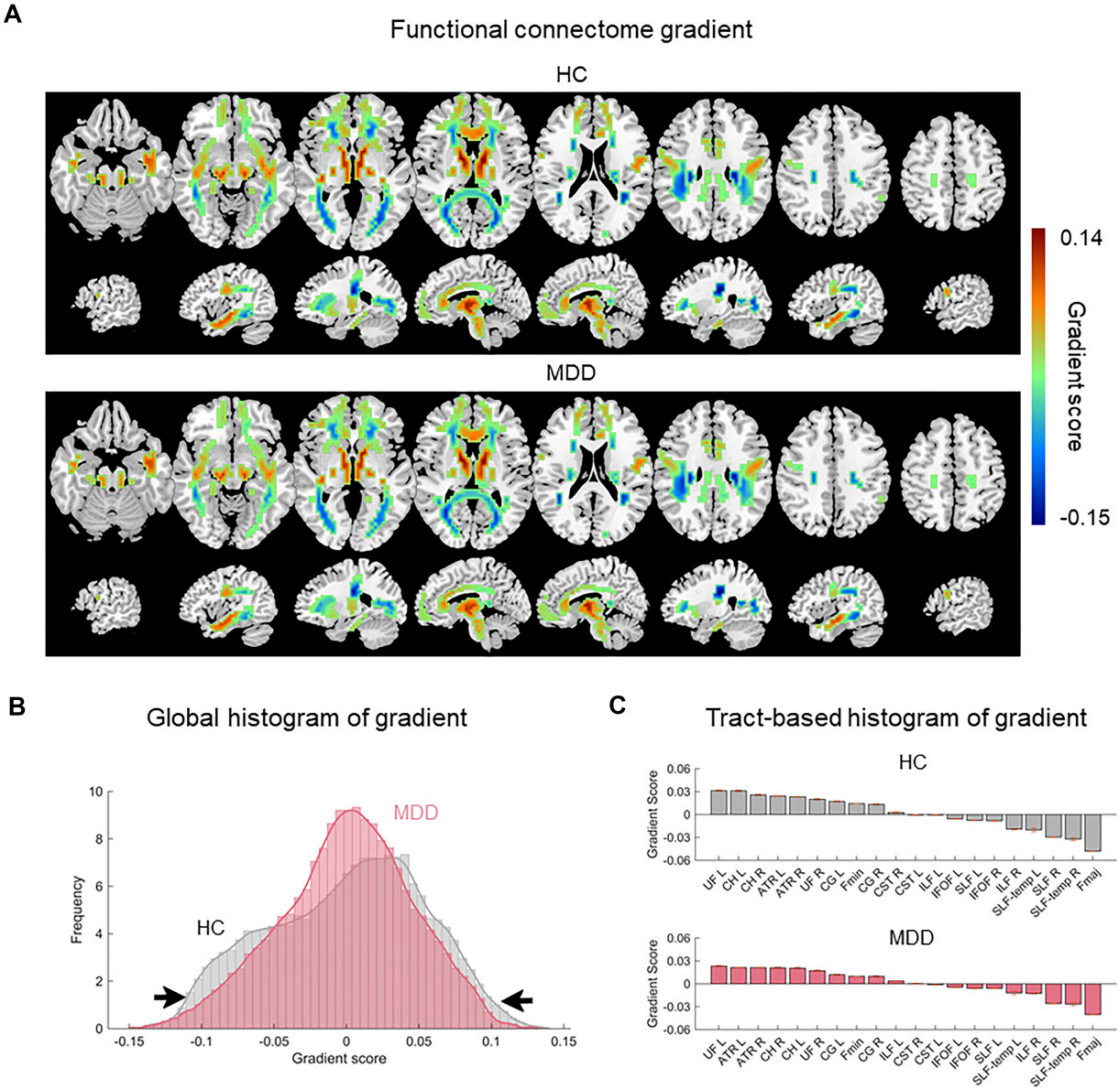
WM functional gradient mapping in HC and patients with MDD. (**A**) In both HC and MDD groups, the principal WM functional gradient was organized along a continuous axis extending from the forceps major and superior longitudinal fasciculus to uncinate fasciculus and anterior thalamic radiation, exhibiting a superficial-to-deep WM pattern. (**B**) Global and (**C**) tract-based histograms showing that the extreme values were contracted in patients with MDD relative to HC. WM, white matter; HC, healthy controls; MDD, major depressive disorder; ATR, anterior thalamic radiation; UF, uncinate fasciculus; CST, corticospinal tract; IFOF, inferior fronto-occipital fasciculus; CG, cingulum (cingulate gyrus); CH, cingulum (hippocampus); ILF, inferior longitudinal fasciculus; SLF, superior longitudinal fasciculus; Fmin, forceps minor; Fmaj, forceps major; L, left; R, right.

The principal WM connectome gradient accounted for 14.6 ± 4.9% of the total connectome variance across all individuals (MDD: 13.3 ± 4.3%; HC: 14.9 ± 5.2%, Figs 1A and S3). This gradient represented a continuous axis extending from the forceps major (Fmaj) and SLF to uncinate fasciculus (UF) and ATR, exhibiting a superficial-to-deep WM pattern (Fig. 1A). The spatial patterns of the group-averaged principal gradient maps were highly similar between MDD patients and HC (*r *= 0.999, *P *< 0.0001). However, histogram inspection revealed that the extremes of the superficial-to-deep gradient were contracted in MDD compared to HC (Fig. 1B and C). Results for the spatial patterns of the second and third gradients are presented in Figs S4 and S5.

### Alterations of WM functional gradients in patients with MDD

**Figure 2:**
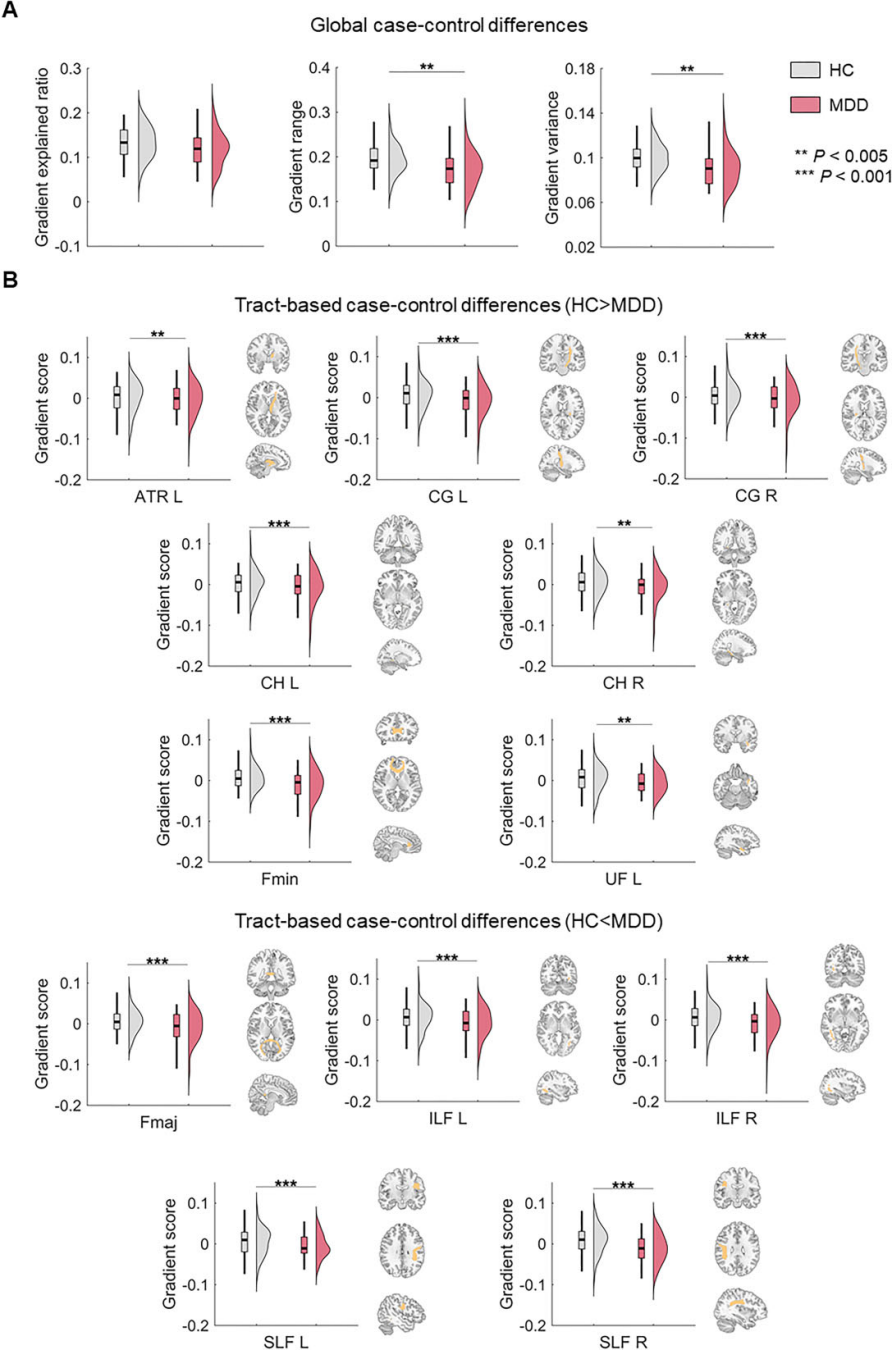
Statistical comparison of the gradient metrics. (**A**) Case-control differences in the global gradient metrics of the principal gradient. (**B**) Case-control differences in the tract-specific gradient scores of the principal gradient. HC, healthy controls; MDD, major depressive disorder; ATR, anterior thalamic radiation; CG, cingulum (cingulate gyrus); CH, cingulum (hippocampus); Fmaj, forceps major; Fmin, forceps minor; ILF, inferior longitudinal fasciculus; SLF, superior longitudinal fasciculus; L, left; R, right. **P < 0.005, ***P < 0.001.

Between-group comparisons revealed that the superficial-to-deep gradient in MDD patients did not significantly differ in the explained ratio compared to HC (Cohen's *d* = −0.41, *P* = 0.098) but showed a narrower range (Cohen's *d* = −0.66, *P* = 0.001, FDR *q *< 0.05) and lower spatial variation (Cohen's *d* = −0.65, *P* < 0.001, FDR *q *< 0.05, Fig. 2A and Table S3). These findings indicate a contracted WM connectome hierarchy in MDD. At the tract-specific level, patients with MDD exhibited lower gradient scores in the left ATR, CG, cingulum hippocampus (CH), forceps minor (Fmin), and left UF. Conversely, they showed higher gradient scores in the Fmaj, as well as the bilateral inferior longitudinal fasciculus (ILF) and SLF, compared to HC (|Cohen's *d*| *>* 0.29, *P* < 0.001, FDR *q *< 0.05, Fig. 2B and Table S4). These findings suggested a less differentiated connectivity pattern in these WM tracts in MDD patients. Comparisons for the second and third gradients are provided in Figs S6 and S7 and Tables S5 and  S6.

### Clinical relevance of WM functional gradients in MDD

**Figure 3:**
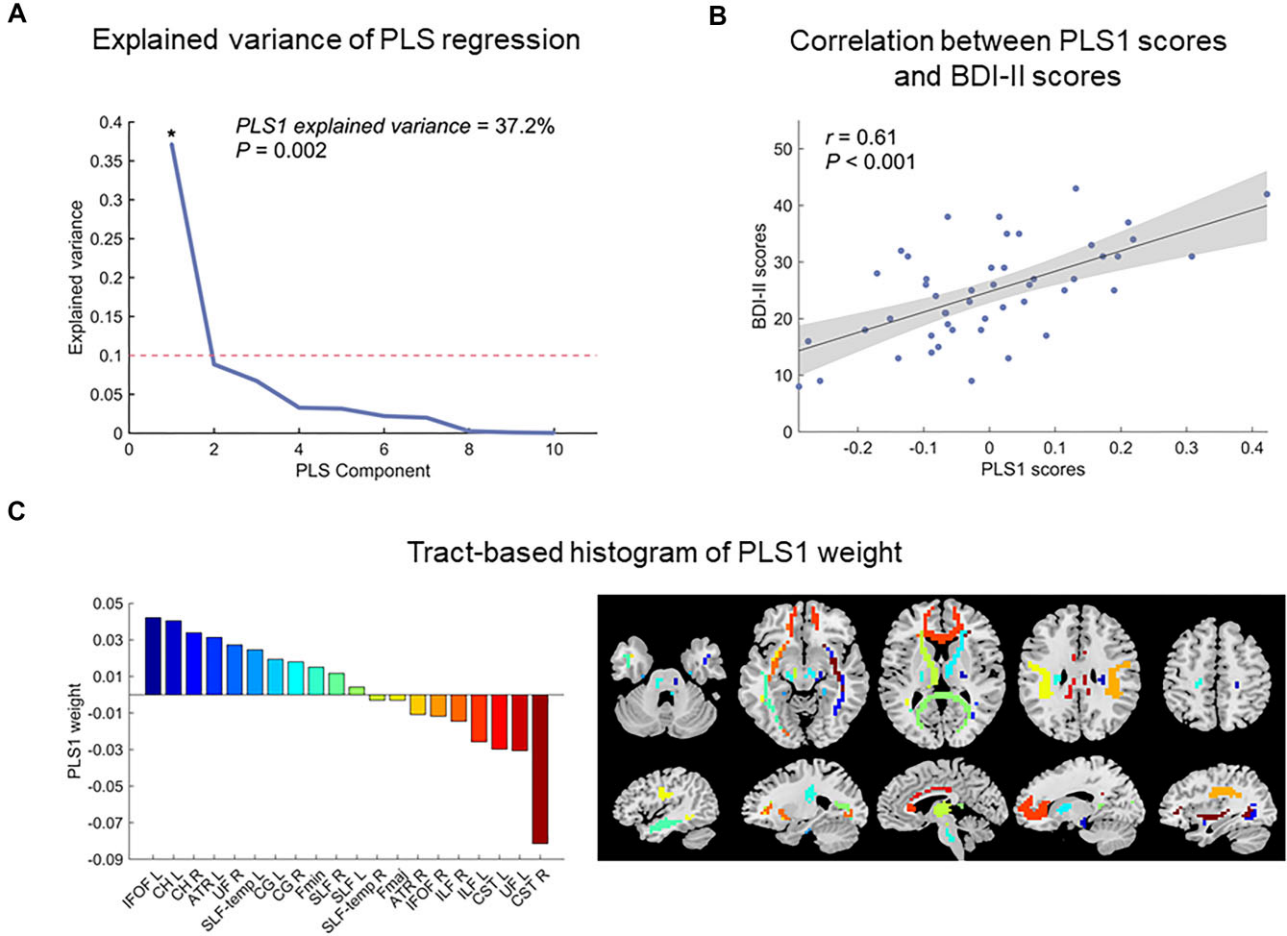
Association between tract-specific gradient scores and clinical symptom severity in MDD. (**A**) Explained ratios for the first 10 components obtained from the PLS regression analysis, with the significant PLS component marked by an asterisk (10 000 permutation tests). (**B**) The PLS1 scores are positively correlated with the BDI-II scores (10 000 permutation tests). Each dot corresponds to a patient. The shaded area represents the 95% confidence intervals. (**C**) Ranked tract weights of PLS1. The weights reflect the relative importance of each tract in explaining the variance in clinical symptom severity, as measured by BDI-II scores. PLS, partial least squares; BDI-II, Beck Depression Inventory-Second Edition; CG, cingulum (cingulate gyrus); ILF, inferior longitudinal fasciculus; CH, cingulum (hippocampus); Fmaj, forceps major; Fmin, forceps minor; SLF, superior longitudinal fasciculus; ATR, anterior thalamic radiation; UF, uncinate fasciculus; CST, corticospinal tract; IFOF, inferior fronto-occipital fasciculus; L, left; R, right.

No significant correlations were observed between global superficial-to-deep gradient metrics and BDI-II scores in MDD patients. However, at the tract-specific level, the first component of the PLS regression (PLS1) explained 37.2% of the variance in BDI-II scores (*P*_perm_ = 0.002, Fig. 3A). The PLS1 scores were significantly positively correlated with BDI-II scores in patients with MDD (*r *= 0.61, *P *< 0.001, Fig. 3B). The strongest positive contributions were observed in the left inferior fronto-occipital fasciculus (IFOF), CH, and left ATR, while the primary negative contributions originated from the bilateral corticospinal tract (CST), ILF, and left UF (Fig. 3C).

### Validation results

Overall, the findings reported above were generally reproducible across different analytical choices (Supplement). (i) Effect of head motion: no significant correlations were observed between mean FD and global gradient metrics (all *r* < 1.0 × 10^−16^, all *P* > 0.05, Fig. S8 and Table S7). Furthermore, analyses in a highly motion-controlled subsample (MDD: *n* = 30, HC: *n* = 33; 54.3% of the original cohort, Table S8) confirmed the principal superficial-to-deep WM gradient hierarchy in MDD (mean *r *= 0.80, *P* < 1.0 × 10^−16^, Table S9). The global metrics revealed that the extremes of the principle gradient were also contracted in MDD compared to HC (Fig. S9). Notably, 58% of the WM tracts identified as significantly altered in MDD in our primary analysis remained detectable in this motion-controlled subsample, including the ATR, CH, Fmin, Fmaj, ILF, and SLF (|Cohen's *d*| > 0.19, *P *< 0.013, FDR *q* < 0.05, Fig. S9 and Table S10). (ii) Different sparsity threshold for retaining functional connections: a strong positive correlation was observed, with a mean *r* = 0.92 between the 5% and 10% thresholds, and *r* = 0.98 between the 10% and 15% thresholds (Fig. S10A). Similarly, Pearson correlations of global gradient metrics across thresholds also demonstrated high consistency (*r* = 0.59–0.91, Fig. S10B). (iii) External validation across cohorts: in an independent dataset, the principal gradient pattern exhibited a similar superficial-to-deep organization (mean *r* = 0.62, *P* < 1.0 × 10^−16^, Table S11) consistent with our main findings. Notably, 75% of the WM tracts identified as significantly altered in MDD in our primary analysis remained detectable in this external dataset, including the CG, CH, Fmin, Fmaj, ILF, and SLF (|Cohen's *d*| > 0.21, *P *< 0.022, FDR *q* < 0.05, Fig. S11 and Table S12). For gradient 2, the spatial organization pattern demonstrated significant similarity to our main findings (mean *r* = 0.15, *P* < 1.0 × 10^−16^). Critically, one-third of MDD-associated WM tract alterations identified in our main analysis (including Fmin, ILF, and SLF) demonstrated reproducible effects in the external validation cohort (|Cohen's *d*| > 0.31, *P* < 0.001, FDR *q* < 0.05, Fig. S12 and Table S13). While gradient 3 did not demonstrate significant spatial concordance with our main findings, 23% of WM tracts exhibiting significant group differences in the main analysis showed reproducible alterations in the external cohort (e.g. ATR, Fmin, and SFL, |Cohen's *d*| > 0.30, *P* < 1.0 × 10^−16^, FDR *q* < 0.05, Fig. S13 and Table S14).

## Discussion

In this study, we highlight a hierarchical dysfunction in the WM functional connectome among patients with MDD. Specifically, we identified a principal superficial-to-deep gradient in WM functional networks in both MDD patients and HC. Compared to HC, MDD patients exhibited a contracted WM functional connectome hierarchy, with the most pronounced alterations observed in gradient scores of the superficial and deep tracts. Importantly, the gradients of these tracts were closely associated with the severity of clinical symptoms. These findings enhance our understanding of the functional organization of WM and its disruption in MDD, shedding light on the neurobiological mechanisms underlying the clinical manifestations of the disorder.

The superficial-to-deep gradient in WM functional networks, extending from the Fmaj and SLF to the UF and ATR in participants, aligns with prior research, which is supported by a significant correlation between the functional gradient and FA (Zhu *et al*., 2023). This observation suggests that the functional gradient of WM might be organized according to its anatomical hierarchy. Previous studies have further shown that superficial WM networks are closely linked to GM networks, while deep WM networks exhibit relative independence from them (Li *et al*., 2020b). Notably, the superficial-to-deep WM gradient has been associated with a broad range of cognitive functions, including emotion recognition, nonverbal reasoning, and facial memory (Zhu *et al*., 2023). This hierarchical organization may therefore serve as a critical communication bridge between distinct WM and GM networks, supporting a variety of cognitive processes.

We identified a contracted WM functional connectome hierarchy in MDD, as indicated by a narrower range and lower variance of gradient scores across the whole brain. This suggests a less differentiated connectivity pattern, consistent with recent findings of disrupted topological architecture in GM functional networks in MDD (Xia *et al*., 2022). Studies on WM functional connectomes have shown a shift towards randomization in MDD (Li *et al*., 2020a). The downgraded connectome hierarchy observed here is supported by these prior reports, where such randomization disrupts the balance between integration and segregation in healthy brain networks, leading to miswired connections. Consequently, this increases the likelihood of incomplete or redundant pathways for information processing, resulting in a less prioritized hierarchical structure in the functional connectome in MDD.

The altered connectome hierarchy reflects disconnections across a broad set of WM fiber tracts, notably in the ATR, CG, UF, Fmaj, Fmin, SLF, and ILF. The ATR, a critical tract connecting the anterior and midline thalamic nuclei with the frontal lobe, plays a key role in cognitive, emotion, and reward processing. DTI studies indicate that microstructural abnormalities in the ATR in MDD might impair cognitive functions and disrupt mood regulation via imbalances in the reward–punishment circuit and affective states (Coenen *et al*., 2012; Lai and Wu, 2014). Furthermore, microstructural abnormalities in both the ATR and the Fmin, a segment of the CC linking the two frontal lobes, have been specifically correlated with anhedonia symptoms in MDD (Pfarr *et al*., 2021). The SLF, which connects the frontal, occipital, parietal, and temporal lobes, is involved in higher-order multi-sensory processing, executive function, and emotional regulation (Lai and Wu, 2014). Decreased FA in the SLF, alongside reduced regional homogeneity of the WM BOLD signal, has been reported in MDD (Huang *et al*., 2024; Ji *et al*., 2023), potentially contributing to deficits in these cognitive and emotional domains. The CG, projecting from the cingulate gyrus to the entorhinal cortex, facilitates communication within the limbic system, affecting emotion, pain perception, and episodic memory (Bubb *et al*., 2018). Decreased WM functional connectivity in the CG has been found in MDD patients, with increased connectivity in this region being associated with better treatment outcomes (Zhang *et al*., 2021). The UF, connecting regions of the limbic system such as the temporal pole, anterior parahippocampus, and amygdala with the orbitofrontal cortex, contributes to dysregulated emotion processing in MDD (Xu *et al*., 2023). These changes in the ATR, Fmin, SLF, and CG align with abnormalities in the limbic–cortical–striatal–thalamic circuit, a key pathway regulating cognition and emotion in MDD (Chen *et al*., 2023; Radoeva *et al*., 2023; Sheline, 2000). Beyond tracts associated with higher-order cognitive functions, MDD also involves abnormalities in tracts linked to primary sensorimotor and visual systems. Specifically, the Fmaj, connecting the posterior occipital lobes, and the ILF, a major occipitotemporal association tract, are implicated in WM functional connectome alterations. Such disruptions in sensorimotor and visual networks have been associated with psychomotor agitation or retardation and visual–emotional deficits in MDD patients (Zhao *et al*., 2021). In conclusion, we have identified a spectrum of tract abnormalities following the superior-to-deep gradient, extending research focus from localized tract changes to a comprehensive understanding of global organizational disruptions. This shift highlights how both primary and higher-order cognitive functions in MDD patients are impacted by functional alterations in WM connectivity.

Disruptions in WM functional organization have been identified across various neuropsychiatric disorders, including schizophrenia (Fan *et al*., 2020; Ji *et al*., 2023; Jiang *et al*., 2019), bipolar disorder (Ji *et al*., 2023), obsessive–compulsive disorder (Ji *et al*., 2023), attention-deficit/hyperactivity disorder (Bu *et al*., 2022), Parkinson's disease (Ji *et al*., 2019; Meng *et al*., 2022; Wang *et al*., 2022), Alzheimer's disease (Zhao *et al*., 2019), and neuromyelitis optica spectrum disorder (Wan *et al*., 2024). Given the overlapping clinical symptoms among MDD, schizophrenia, and bipolar disorder, identifying both shared and disorder-specific abnormalities in psychiatric connectomes is crucial for understanding their distinct neural mechanisms (Xia *et al*., 2019). A transdiagnostic study reported increased amplitude of low-frequency fluctuation (ALFF) in the ATR in schizophrenia and bipolar disorder, as well as altered ALFF in the SLF specific to bipolar disorder, with no significant changes observed in MDD (Ji *et al*., 2023). Additionally, another study observed reduced regional homogeneity of the WM BOLD signal in the SLF in MDD (Huang *et al*., 2024). In contrast to previous findings, our study identified altered gradient scores in both the ATR and SLF in MDD, suggesting that ATR dysfunction may be a shared feature across MDD, schizophrenia, and bipolar disorder, while SLF abnormalities might represent a common neural substrate between MDD and bipolar disorder. Our findings suggest that gradient-based analyses can uncover more widespread WM functional abnormalities in MDD, including alterations in the CG, Fmaj, Fmin, and ILF, which may have been overlooked in previous studies focusing on localized functional activity. By capturing disruptions in large-scale functional organization, this approach provides a more comprehensive framework for understanding the neurophysiological basis of MDD.

Moreover, our results suggest that specific WM connectivity patterns, rather than global network organization, are associated with the clinical manifestation of MDD. The tracts that contributed most significantly to the correlation with BDI-II scores in our study are located in regions we identified as abnormal in MDD, including the CH, ILF, ATR, and UF. These tracts are essential for sensory integration and emotional regulation, and their disruptions have been strongly linked to clinical features of MDD, such as depressive severity, illness duration, and the number of depressive episodes (Lu *et al*., 2021; Zhang *et al*., 2022, 2021; Zhao *et al*., 2021). Additionally, we observed positive contributions from the IFOF and negative contributions from the CST. The IFOF, which connects the frontal lobe with the occipital and temporal lobes, plays a key role in integrating frontal lobe-related inhibitory control and occipital lobe-related sensory processing (Zhang *et al*., 2022). The CST, a motor pathway that connects the cerebral cortex to lower motor neurons in the spinal cord, controls limb and trunk movements. Abnormalities in the microstructure of these regions has been associated with disrupted sensory integration and impaired cognitive inhibition toward sensory stimulus and emotion, contributing to clinical symptoms such as suicidal ideation and behavior in MDD (Pfarr *et al*., 2021; Zhang *et al*., 2022). Together, these findings emphasize how disruptions in the hierarchical structure of WM functional networks contribute to the clinical manifestations of MDD, potentially serving as biomarkers for symptom evaluation.

In addition to the principal superficial-to-deep gradient, which probably reflects the anatomical foundation of WM functional organization, the second and third gradients provide complementary insights into WM functional disruptions in MDD. The second gradient appears to represent a top-down functional architecture, with one end encompassing the SLF and CG, which primarily connect parietal and cingulate regions, and the other end located in the SLF-temp, ILF, and Fmin, associated with occipitotemporal integration and frontal processing (Bubb *et al*., 2018; Lai and Wu, 2014; Zhao *et al*., 2021). Compared to the principal gradient, abnormalities in this second gradient are less pronounced in MDD, suggesting a relatively preserved hierarchical organization along this axis. In contrast, the third gradient follows a frontal-to-occipital organization, with one extreme in the Fmin and CH, and the other in the Fmaj and SLF. This gradient shows more significant alterations in MDD, possibly reflecting disruptions in hierarchical functional processing from higher-order cognitive regions to more primary visual and auditory processing areas. Notably, shared alterations across gradients are observed in key tracts, including the ATR, Fmin, ILF, UF, and SLF, suggesting widespread yet distinct patterns of WM dysfunction in MDD across different functional domains. Collectively, these findings underscore the importance of gradient-based analyses in uncovering multi-dimensional disruptions in WM organization in MDD.

Several issues of the current study need to be further addressed. First, this study examined the clinical associations of WM functional gradients using BDI-II scores. Nevertheless, MDD is characterized by a variety of cognitive impairments and is influenced by numerous clinical factors, such as age of onset, medication status, and first-episode status, which were not assessed in this retrospective analysis. Future studies incorporating detailed cognitive and clinical assessments could offer a more comprehensive understanding of the intricate relationship between WM functional gradients and the clinical manifestations of MDD. Furthermore, longitudinal studies that track treatment data could offer valuable insights into the potential clinical relevance of WM functional gradients in monitoring treatment response and outcomes. Second, prior studies have demonstrated a spatiotemporal topological correspondence between BOLD signals and glucose metabolism in brain WM in healthy individuals (Li *et al*., 2023). Further investigations into the relationship between alterations in WM functional gradients and neurometabolic changes could substantially enhance our understanding of the neurobiological underpinnings of the disorder. Third, while our study focused on functional organization alterations in the WM connectome, future research should explore the structural underpinnings of these functional networks and investigate the coupling between functional and structural connectomes. Understanding the relationship between these two domains in MDD could provide a more integrated view of WM abnormalities. Finally, both disrupted functional gradients in WM and GM have been observed in MDD patients. Future studies should examine how these alterations in WM and GM connectomes interact and influence each other. Such investigations could offer deeper insights into the neurobiological mechanisms of MDD and help identify biomarkers for classification and prediction.

## Supplementary Material

kkaf008_Supplemental_File
