# Supplementary material for: White matter functional connectome gradient dysfunction in major depressive disorder
Source: Psychoradiology. 2025 Apr 28;5:kkaf008. doi: 10.1093/psyrad/kkaf008 (PMC12076206; doi:10.1093/psyrad/kkaf008)
Supplement: kkaf008_Supplemental_File [file kkaf008_supplemental_file.docx]

**Supplement**

**Validation Methods and Results**

**(1) Effect of Head Motion**

To further evaluate the potential residual effects of head motion on gradient metrics, we conducted two additional validation analyses.

First, we computed Pearson’s correlation between mean framewise displacement (FD) and global gradient metrics across all participants for the three gradients. The absence of significant correlations between mean FD and global gradient metrics (*r* < 1.0×10⁻¹⁶, *P* > 0.05, Figure S8 and Table S7) demonstrates that head motion artifacts do not systematically confound the observed outcomes.

Furthermore, application of a rigorous mean FD threshold (0.2 mm) to create a motion-controlled subsample resulted in the exclusion of 18 patients and 35 healthy controls (HC) due to excessive motion. The final sample included 33 patients and 30 HCs. Demographic and clinical characteristics showed no significant differences in age, mean FD, maximum translation, or maximum rotation between major depressive disorder (MDD) and HC (all *P* > 0.11), though MDD patients had higher Beck Depression Inventory-Second Edition (BDI-II) scores (*P* < 0.001, Table S8). Reanalysis confirmed the principal white matter (WM) connectome gradient accounted for 12.1% ± 3.8% of total variance (MDD: 11.0% ± 3.5%; HC: 13.1% ± 4.1%, Figure S15). This gradient represented a continuous axis extending from the forceps major (Fmaj) and superior longitudinal fasciculus (SLF) to the bilateral anterior thalamic radiation (ATR), exhibiting a superficial-to-deep WM pattern (Figure S14). The spatial patterns of the group-averaged principal gradient maps were highly similar between MDD patients and HC (*r* = 0.99, *P* < 0.0001). Histogram inspection revealed that the extremes of the superficial-to-deep gradient were contracted in MDD compared to HC (Figure S14). Results for the spatial patterns of the second and third gradients are presented in Figures S16 and S17. Between-group comparisons revealed that the superficial-to-deep gradient in MDD patients showed a lower explained ratio (Cohen’s *d* = -0.51, *P* = 0.049, FDR *q* < 0.05), a narrower range (Cohen’s *d* = -0.62, *P* = 0.016, FDR *q* < 0.05), and lower spatial variation (Cohen’s *d*=-0.57, *P* = 0.027, FDR *q* < 0.05, Figure S9 and Table S15). These findings indicate a contracted WM connectome hierarchy in MDD. At the tract-specific level, patients with MDD exhibited lower gradient scores in the bilateral ATR, the right corticospinal tract, the bilateral cingulum hippocampus and forceps minor. Conversely, they showed higher gradient scores in the Fmaj, the left inferior longitudinal fasciculus, as well as the left SLF, compared to HC (|Cohen’s *d*| > 0.19, *P* < 0.013, FDR *q* < 0.05, Figure S9 and Table S10). These findings suggested a less differentiated connectivity pattern in these WM tracts in MDD patients. Comparison for the second and third gradients are provided in Figures S18-S19 and Tables S16-S17.

**(2) Different Sparsity Threshold for Retaining Functional Connections**

To evaluate the reliability of our results, we computed WM functional gradients using sparsity thresholds of 5% and 15%. First, we observed a strong positive correlation among gradient maps, with a mean *r* = 0.92 between the 5% and 10% threshold, and *r* = 0.98 between the 10% and 15% thresholds were observed (Figure S10A). Second, all three global gradient metrics demonstrated strong inter-threshold consistency between the tested thresholds and the main results. This was evident in the explained variance ratio (5% and 10%: *r* = 0.59, *P* < 1.0×10^-16^; 15% and 10%: *r* = 0.80, *P* < 1.0×10^-16^), the gradient range (5% and 10%: *r* = 0. 61, *P* < 1.0×10^-16^; 10% and 15%: *r* = 0. 89, *P* < 1.0×10^-16^), and the gradient variance (5% and 10%: *r* = 0. 77, *P* < 1.0×10^-16^; 10% and 15%: *r* = 0. 91, *P* < 1.0×10^-16^) (Figure S10B).

**(3) External Validation Across Cohorts**

To evaluate the generalizability of our findings, we conducted an external validation in an independent cohort comprising 29 MDD patients and 27 HCs from the Hiroshima Kajikawa Hospital (Table S2). Participants were scanned on a Siemens Symphon 3.0T scanner (12-channel head coil) using an echo-planar imaging sequence with the following parameters: repetition time (TR) = 2700 ms, echo time (TE) = 31 ms, flip angle (FA) = 90°, matrix = 64×64, slice thickness = 3 mm, and 38 axial slices. After excluding 4 patients and 2 HCs due to excessive motion (maximum translation >3 mm, rotation >3°, mean FD >0.3 mm, or >50% scrubbed volumes), the final sample included 29 patients and 27 HCs. The HKH dataset underwent identical data preprocessing procedures to those implemented in the main methodology section, ensuring methodological consistency and reproducibility of experimental results.

Demographic characteristics showed no significant differences in age, sex, mean FD, maximum translation, or maximum rotation between patients with MDD and HC (all *P* > 0.06). However, patients with MDD exhibited significantly higher BDI-II scores compared to the HC group (*P* < 0.001, Table S2).

Consistent with our main findings, the principal gradient pattern in this independent dataset also exhibited a similar superficial-to-deep organization (mean *r* = 0.62, *P* < 1.0×10^-16^, Table S11). Specifically, the principal WM connectome gradient accounted for 14.2% ± 5.0% of the total connectome variance across all individuals (MDD: 13.5% ± 4.7%; HC: 14.9% ±5.3%, Figure S21). This gradient represented a continuous axis extending from the Fmaj and SLF to cingulate gyrus, exhibiting a superficial-to-deep WM pattern (Figure S20). The spatial patterns of the group-averaged principal gradient maps were highly similar between MDD patients and HC (*r* = 0.99, *P* < 0.0001). Histogram inspection revealed that the extremes of the superficial-to-deep gradient were contracted in MDD compared to HC (Figure S20). For gradient 2, the spatial organization pattern demonstrated significant similarity to our main findings (mean *r* = 0.15, *P* < 1.0×10^-16^). Gradient 3 did not demonstrate significant spatial concordance with our main findings. Results for the spatial patterns of the second and third gradients are presented in Figures S22 and S23.

Between-group comparisons demonstrated no significant differences in global gradient metrics (explained ratio: Cohen’s *d* = -0.32, *P* = 0.232; range: Cohen’s *d* = -0.39, *P* = 0.154; spatial variation: Cohen’s *d* = -0.44, *P* = 0.107, Table S18). However, tract-specific alterations were replicated: MDD patients showed lower gradient scores in bilateral corticospinal tract, cingulate gyrus, cingulum hippocampus, and forceps minor, alongside higher scores in Fmaj, right inferior fronto-occipital fasciculus (IFOF), bilateral inferior longitudinal fasciculus, and left SLF (|Cohen’s d| > 0.21, P < 0.022, FDR q < 0.05; Figure S11, Table S12). Critically in gradient 2, one-third of MDD-associated white matter tract alterations identified in our main analysis (including Fmin, ILF, and SLF) demonstrated reproducible effects in the external validation cohort (|Cohen’s d| > 0.31, P < 0.001, FDR q < 0.05; see Figure S12 and Table S13). 23% of gradient 3 white matter tracts exhibiting significant group differences in the main analysis showed reproducible alterations in the external cohort (e.g., ATR, Fmin, SFL, |Cohen’s *d*| > 0.30, *P* < 1.0×10^-16^, FDR *q* < 0.05, Figure S13, Table S14). These findings suggested a less differentiated connectivity pattern in these WM tracts in MDD patients.

**Supplementary Figures**


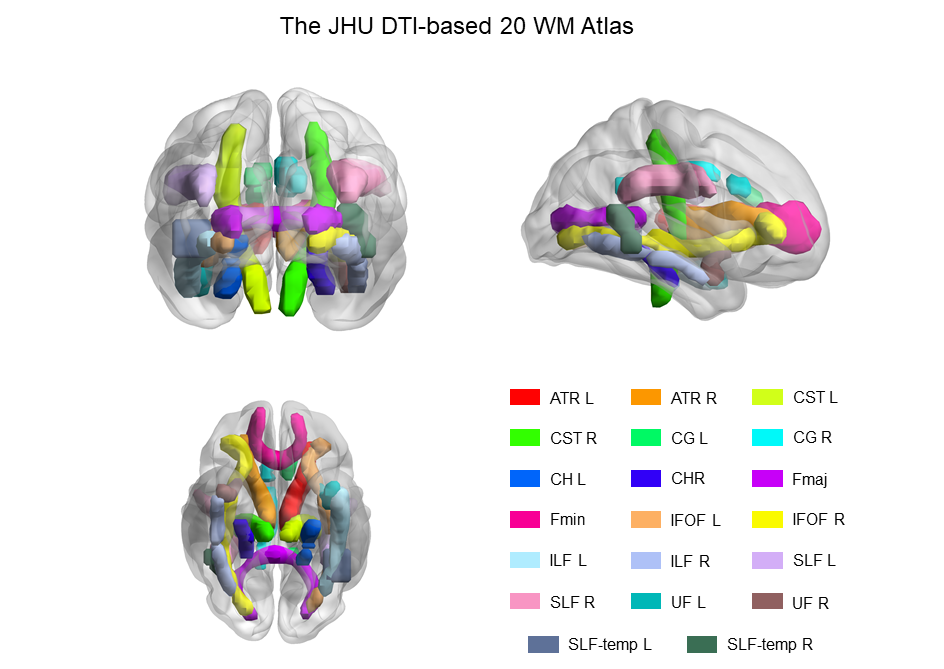


**Supplementary Figure 1.** JHU DTI-based WM atlas. Three-dimensional views (axial, sagittal, and coronal orientations) display 20 distinct WM tracts reconstructed from the JHU DTI-based WM atlas. Each tract is color-coded according to the reference color bar. WM, white matter; JHU DTI, Johns Hopkins University diffusion tensor imaging. ATR, anterior thalamic radiation; CST, corticospinal tract; CG, cingulum (cingulate gyrus); CH, cingulum (hippocampus); Fmaj, forceps major; Fmin, forceps minor; IFOF, inferior fronto-occipital fasciculus; ILF, inferior longitudinal fasciculus; SLF, superior longitudinal fasciculus; UF, uncinate fasciculus; L, left; R, right.


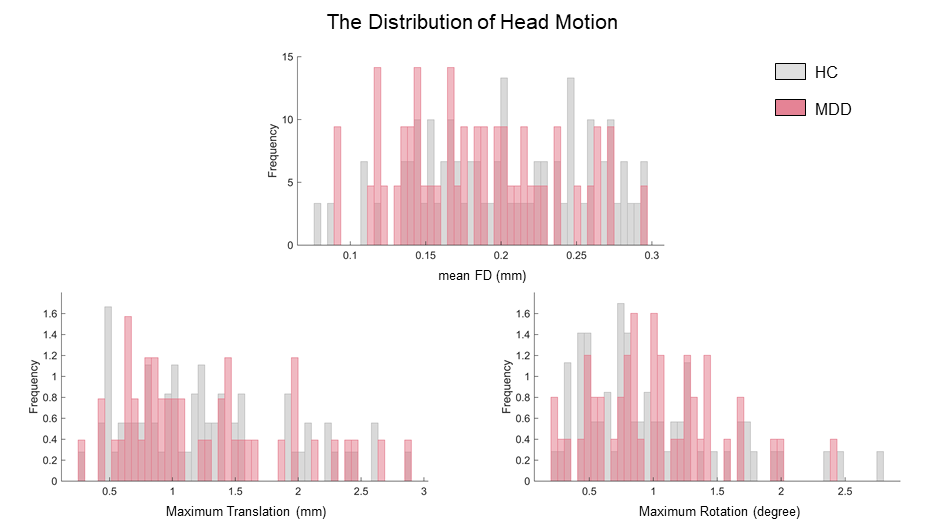


**Supplementary Figure 2.** Head motion parameter distributions across groups. Bar plots show the frequency distributions of mean FD, maximum translation, and maximum rotation (degree) for HC (gray) and MDD patients (red). The y-axis represents frequency counts, and the x-axis indicates the magnitude of each motion parameter. FD, framewise displacement.


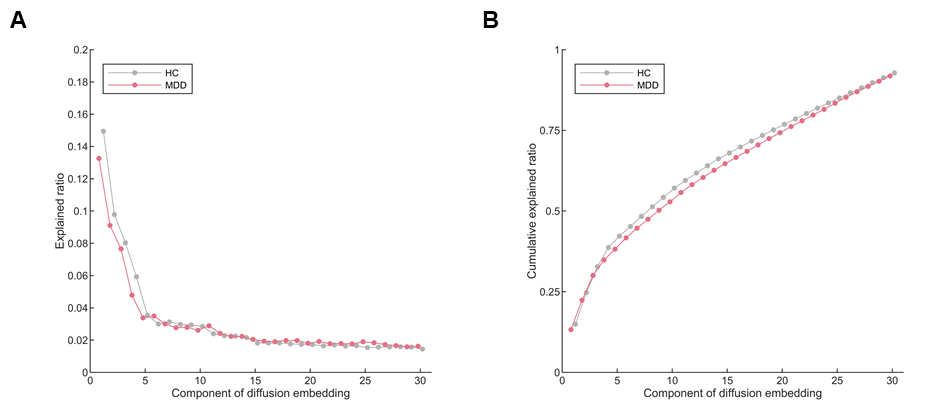


**Supplementary Figure 3.** The explained ratio of the gradients in white matter functional connectome. (A) The averaged explained ratio and (B**)** the cumulative averaged explained ratio of the first 30 diffusion embedding components in the HC and MDD groups. The HC group is shown in gray, and the MDD group is shown in red. The first three gradients explained 14.6% ± 4.9% of the total variance in the connectome across all individuals (MDD, 13.3% ± 4.3%; HC, 14.9% ± 5.2%). HC, healthy controls; MDD, major depressive disorder.


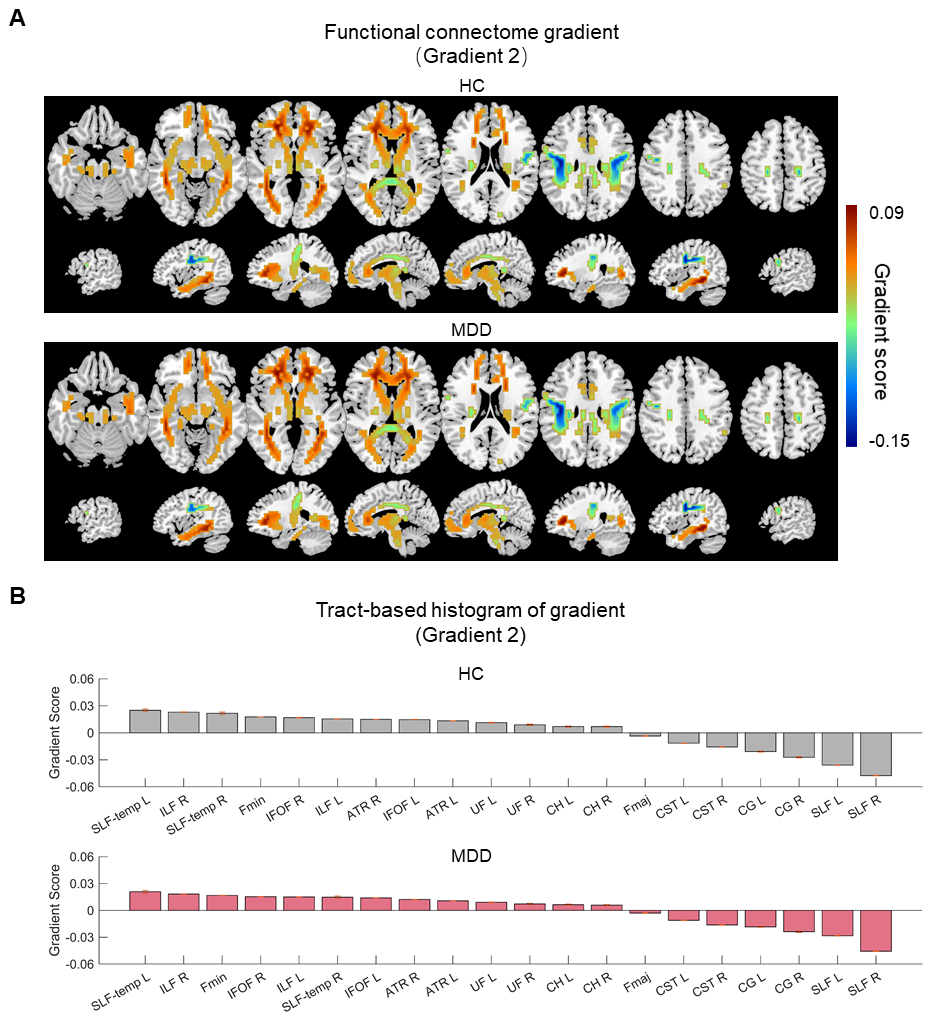


**Supplementary Figure 4.** WM functional gradient 2 in HC and patients with MDD. (A) In both HC and MDD groups, the second WM functional gradient extended between the inferior longitudinal fasciculus and the superior longitudinal fasciculus tracts. (B) Tract-based histograms showing that the extreme values were contracted in patients with MDD relative to HC. WM, white matter; HC, healthy controls; MDD, major depressive disorder; ATR, anterior thalamic radiation; CST, corticospinal tract; CG, cingulum (cingulate gyrus); CH, cingulum (hippocampus); Fmaj, forceps major; Fmin, forceps minor; IFOF, inferior fronto-occipital fasciculus; ILF, inferior longitudinal fasciculus; SLF, superior longitudinal fasciculus; UF, uncinate fasciculus; L, left; R, right.


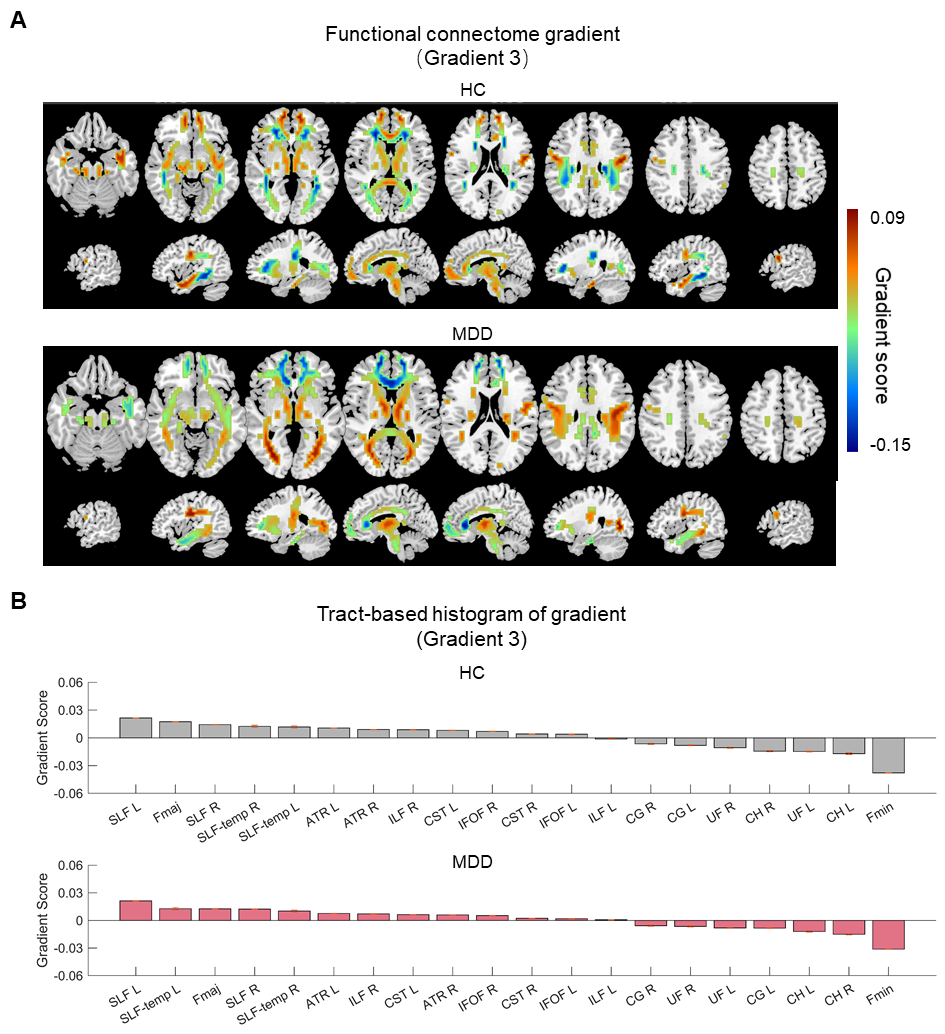


**Supplementary Figure 5.** WM functional gradient 3 in HC and patients with MDD. (A) In both HC and MDD groups, the third WM functional gradient extended between the superior longitudinal fasciculus and the forceps minor tracts. (B) Tract-based histograms showing that the extreme values were contracted in patients with MDD relative to HC. WM, white matter; HC, healthy controls; MDD, major depressive disorder; ATR, anterior thalamic radiation; CST, corticospinal tract; CG, cingulum (cingulate gyrus); CH, cingulum (hippocampus); Fmaj, forceps major; Fmin, forceps minor; IFOF, inferior fronto-occipital fasciculus; ILF, inferior longitudinal fasciculus; SLF, superior longitudinal fasciculus; UF, uncinate fasciculus; L, left; R, right.


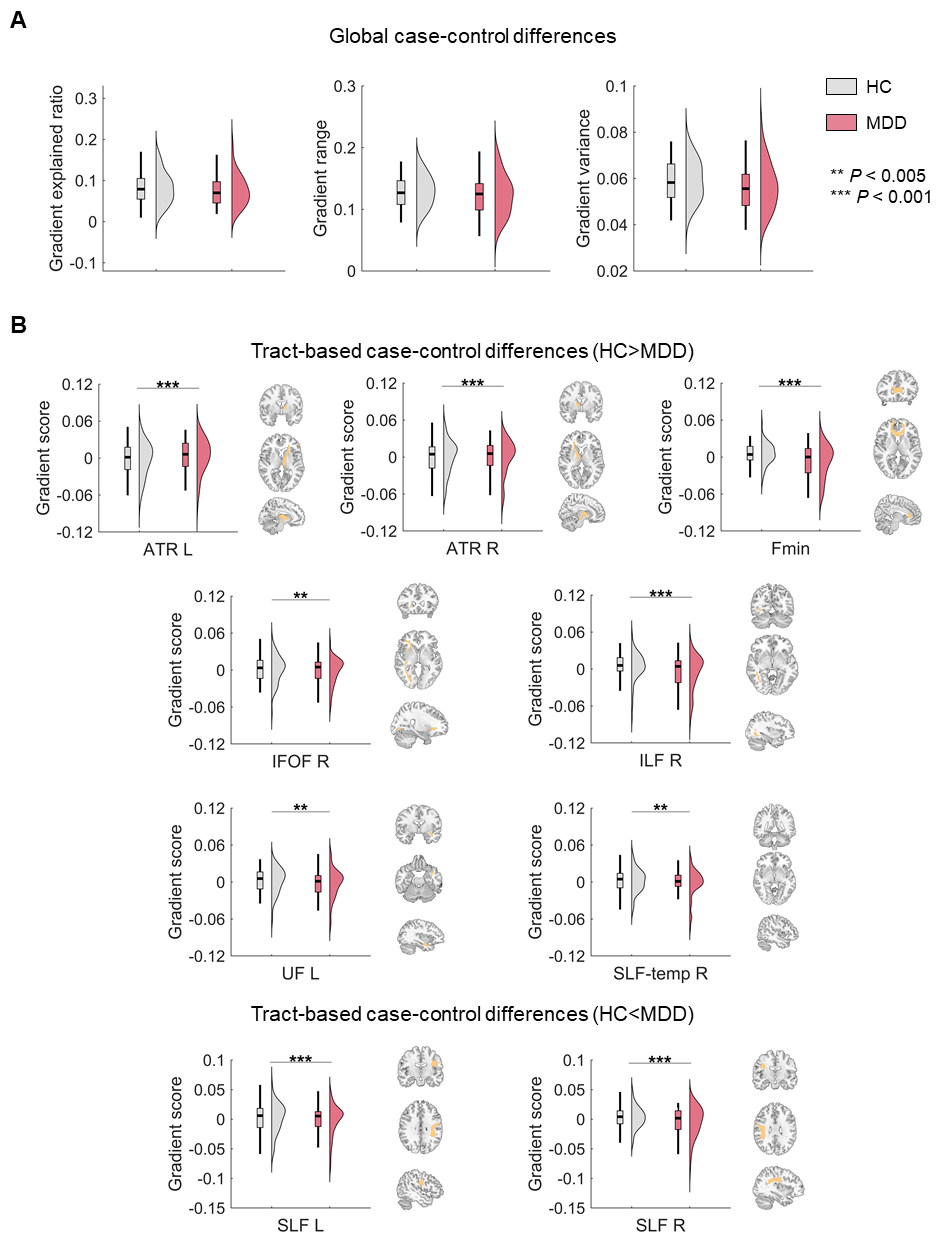


**Supplementary Figure 6.** Statistical comparison of the metrics of gradient 2. (A) Case-control differences in the global gradient metrics of the second gradient. (B) Case-control differences in the tract-specific gradient scores of the second gradient. ATR, anterior thalamic radiation; Fmin, forceps minor; IFOF, inferior fronto-occipital fasciculus; ILF, inferior longitudinal fasciculus; SLF, superior longitudinal fasciculus; UF, uncinate fasciculus; L, left; R, right. ** *P* < 0.005, *** *P* < 0.001.


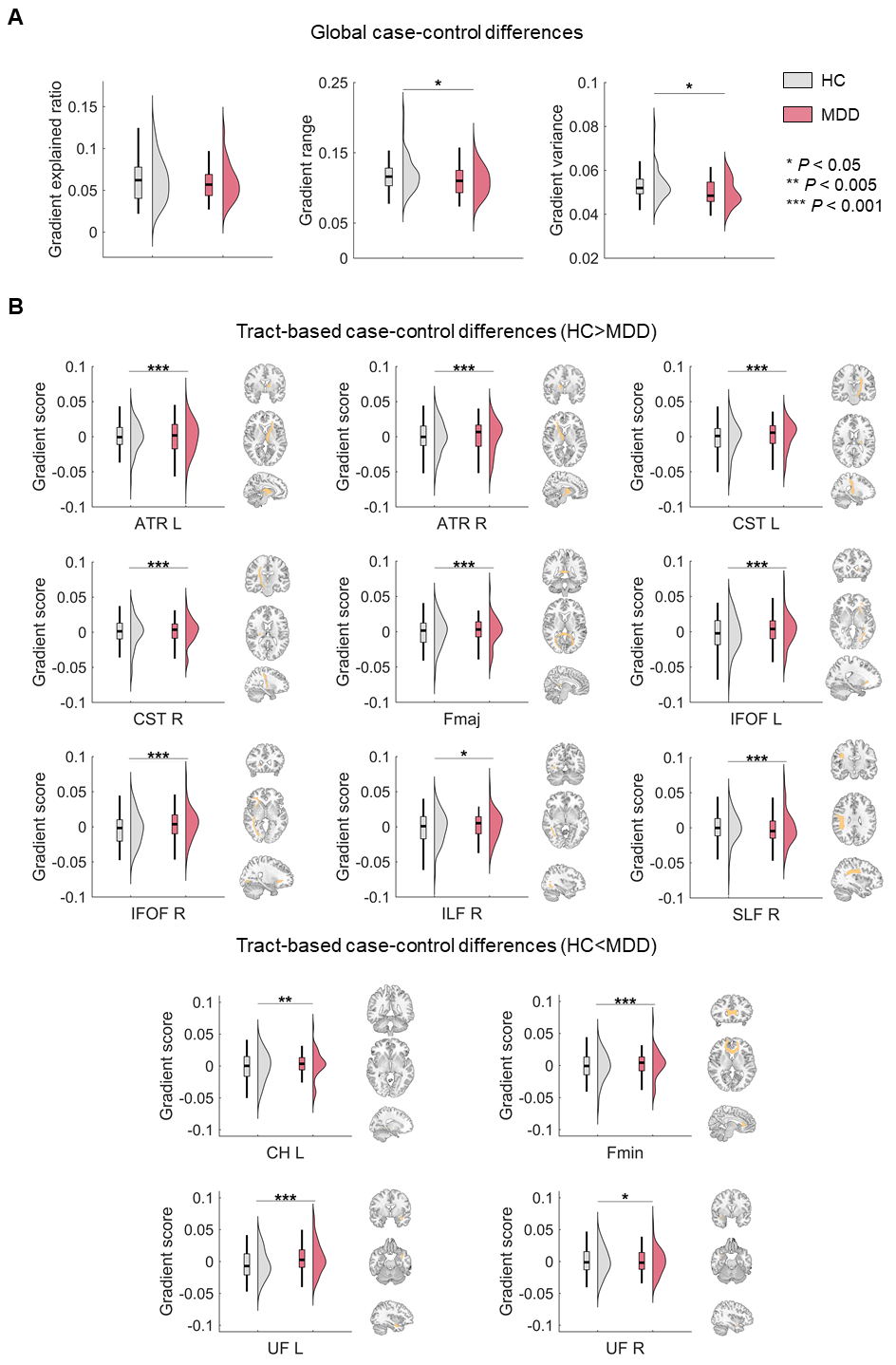


**Supplementary Figure 7.** Statistical comparison of the metrics of gradient 3. (A) Case-control differences in the global gradient metrics of the third gradient. (B) Case-control differences in the tract-specific gradient scores of the third gradient. ATR, anterior thalamic radiation; CST, corticospinal tract; CH, cingulum (hippocampus); Fmaj, forceps major; Fmin, forceps minor; IFOF, inferior fronto-occipital fasciculus; ILF, inferior longitudinal fasciculus; SLF, superior longitudinal fasciculus; UF, uncinate fasciculus; L, left; R, right. * *P* < 0.05, ** *P* < 0.005, *** *P* < 0.001.

**
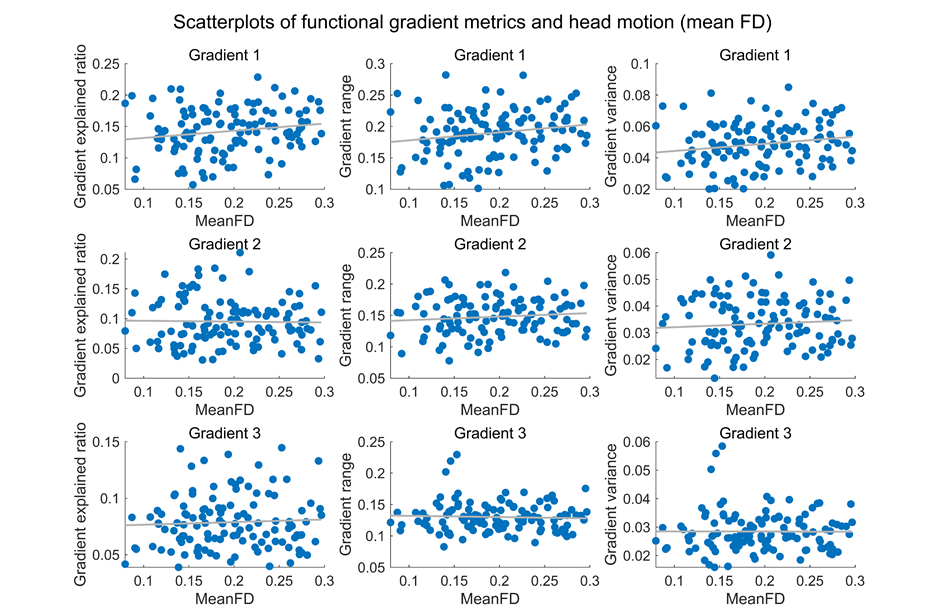
**

**Supplementary Figure 8.** Associations between functional gradient metrics and head motion (mean FD). Each dot corresponds to an individual. FD, framewise displacement.

**
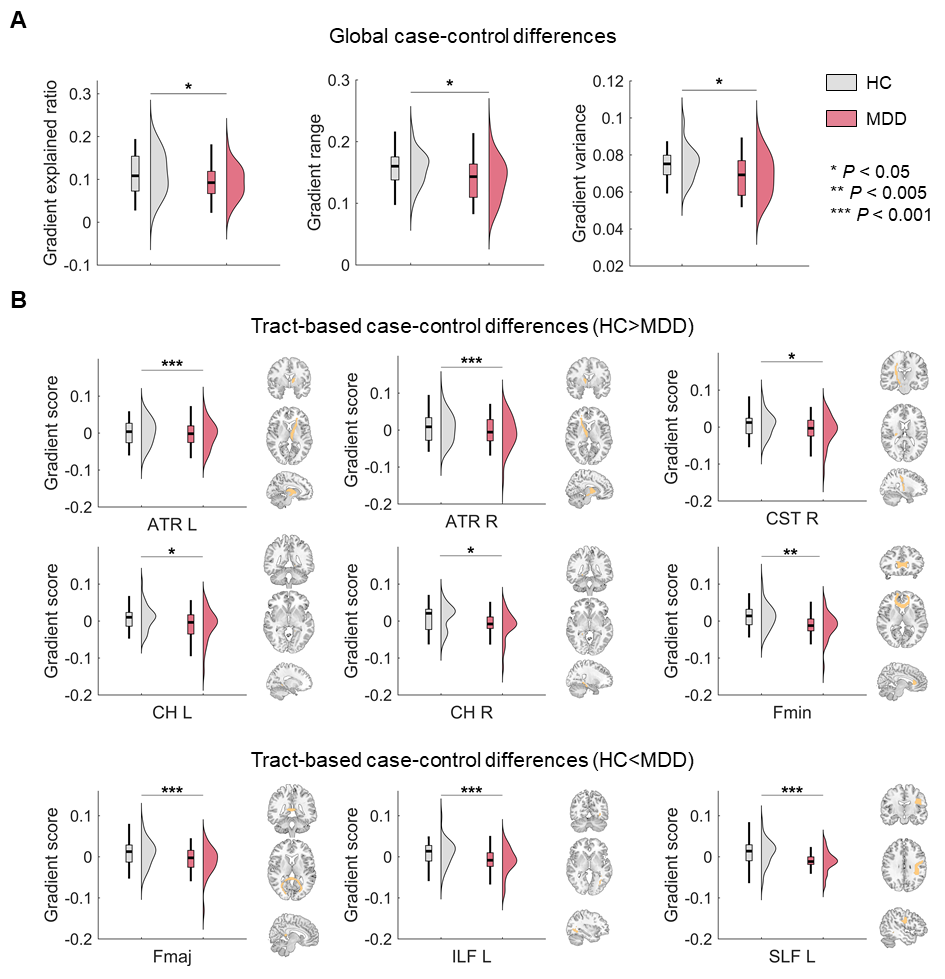
**

**Supplementary Figure 9.** Statistical comparison of the gradient metrics in participants with stricter motion control (mean FD < 0.2 mm). (A) Case-control differences in the global gradient metrics of the principal gradient. (B) Case-control differences in the tract-specific gradient scores of the principal gradient. HC, healthy controls; MDD, major depressive disorder; FD, framewise displacement; ATR, anterior thalamic radiation; CST, corticospinal tract; CH, cingulum (hippocampus); ILF, inferior longitudinal fasciculus; SLF, superior longitudinal fasciculus; Fmin, forceps minor; Fmaj, forceps major; L, left; R, right. * *P* < 0.05, ** *P* < 0.005, *** *P* < 0.001.

**
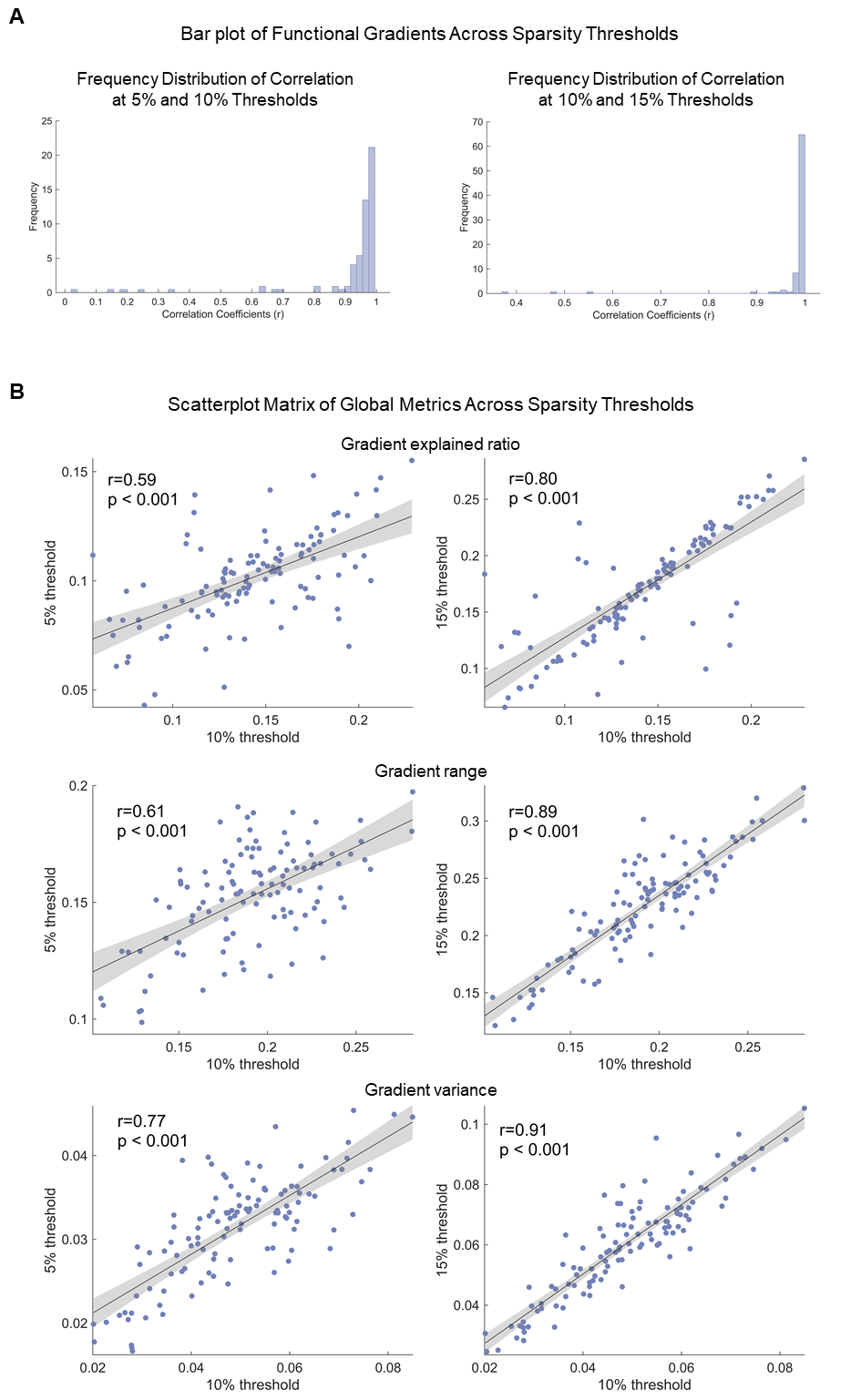
**

**Supplementary Figure 10.** Sensitivity analysis of functional gradients across sparsity thresholds. (A) Correlations between functional gradient maps derived from adjacent thresholds. Left: Distribution of correlations between 5% and 10% sparsity thresholds (x-axis: *r*; y-axis: frequency); Right: Distribution of correlations between 10% and 15% sparsity thresholds. (B) Correlations of global gradient metrics between different thresholds. Each dot corresponds to a patient. The shaded area represents the 95% confidence intervals.

**
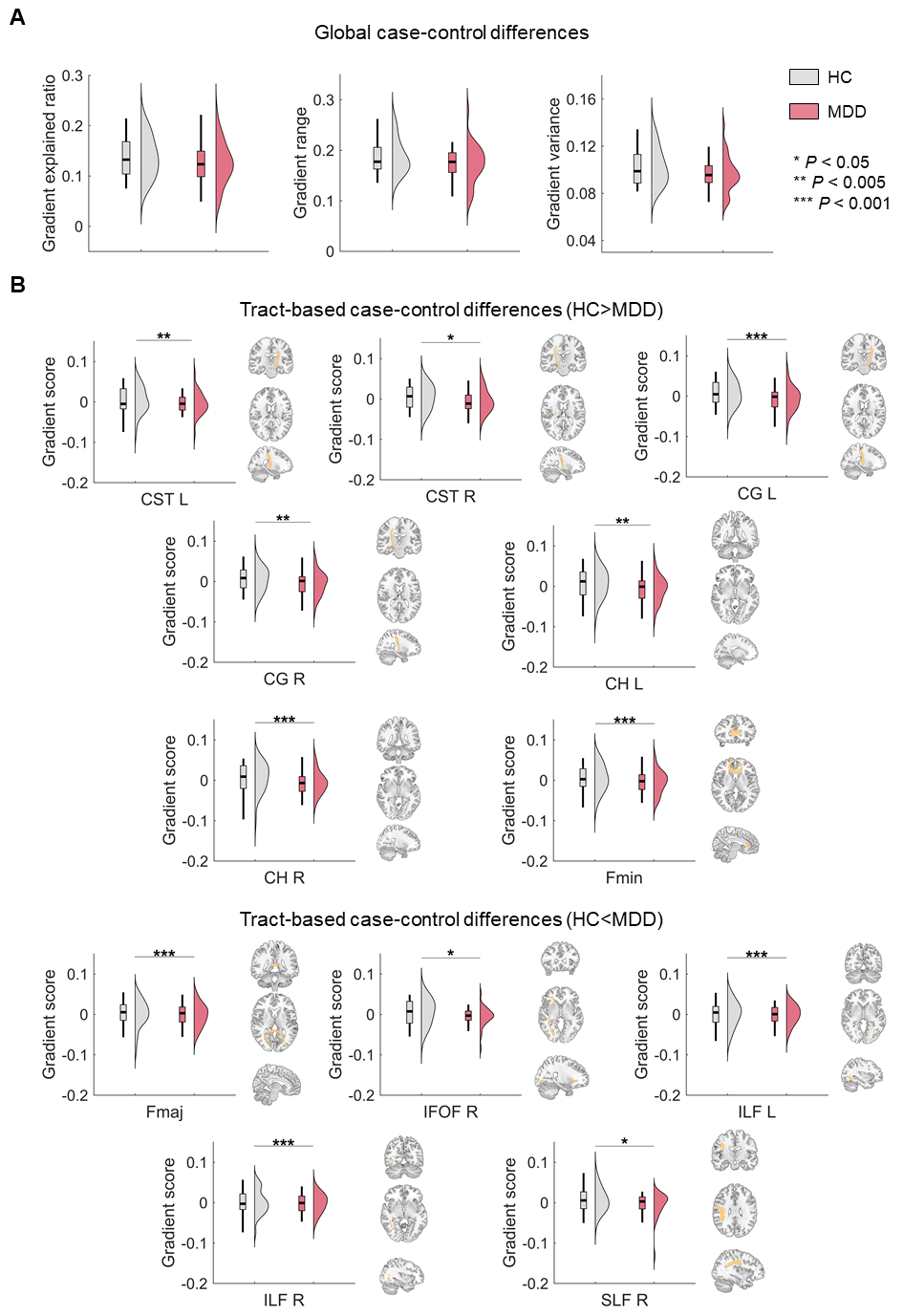
**

**Supplementary Figure 11.** Statistical comparison of the gradient metrics in HKH. (A) Case-control differences in the global gradient metrics of the principal gradient. (B) Case-control differences in the tract-specific gradient scores of the principal gradient. HC, healthy controls; MDD, major depressive disorder; HKH, Hiroshima Kajikawa Hospital; CST, corticospinal tract; IFOF, inferior fronto-occipital fasciculus; CG, cingulum (cingulate gyrus); CH, cingulum (hippocampus); ILF, inferior longitudinal fasciculus; SLF, superior longitudinal fasciculus; Fmin, forceps minor; Fmaj, forceps major; L, left; R, right. * *P* < 0.05, ** *P* < 0.005, *** *P* < 0.001.

**
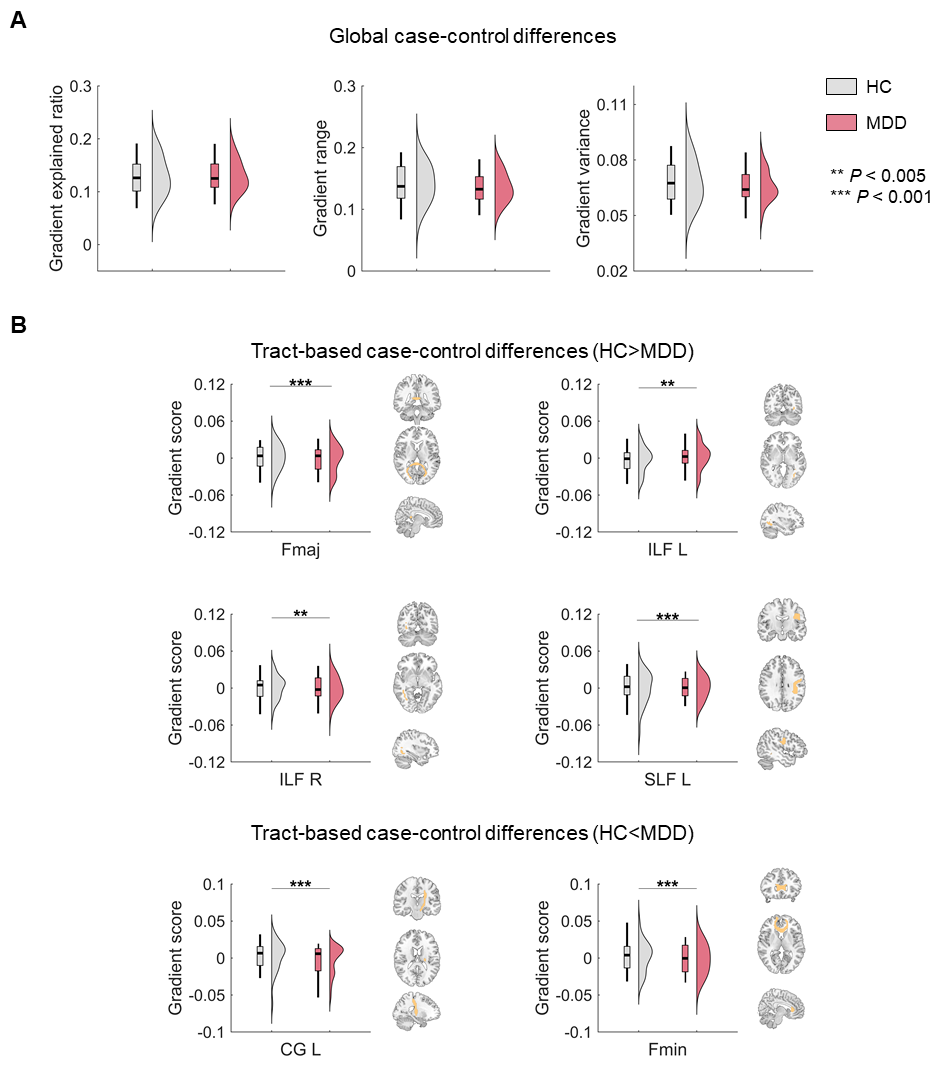
**

**Supplementary Figure 12.** Statistical comparison of the metrics of gradient 2 in HKH. (A) Case-control differences in the global gradient metrics of the second gradient. (B) Case-control differences in the tract-specific gradient scores of the second gradient. HKH, Hiroshima Kajikawa Hospital; CG, cingulum (cingulate gyrus); Fmaj, forceps major; Fmin, forceps minor; ILF, inferior longitudinal fasciculus; SLF, superior longitudinal fasciculus; L, left; R, right. ** *P* < 0.005, *** *P* < 0.001.

**
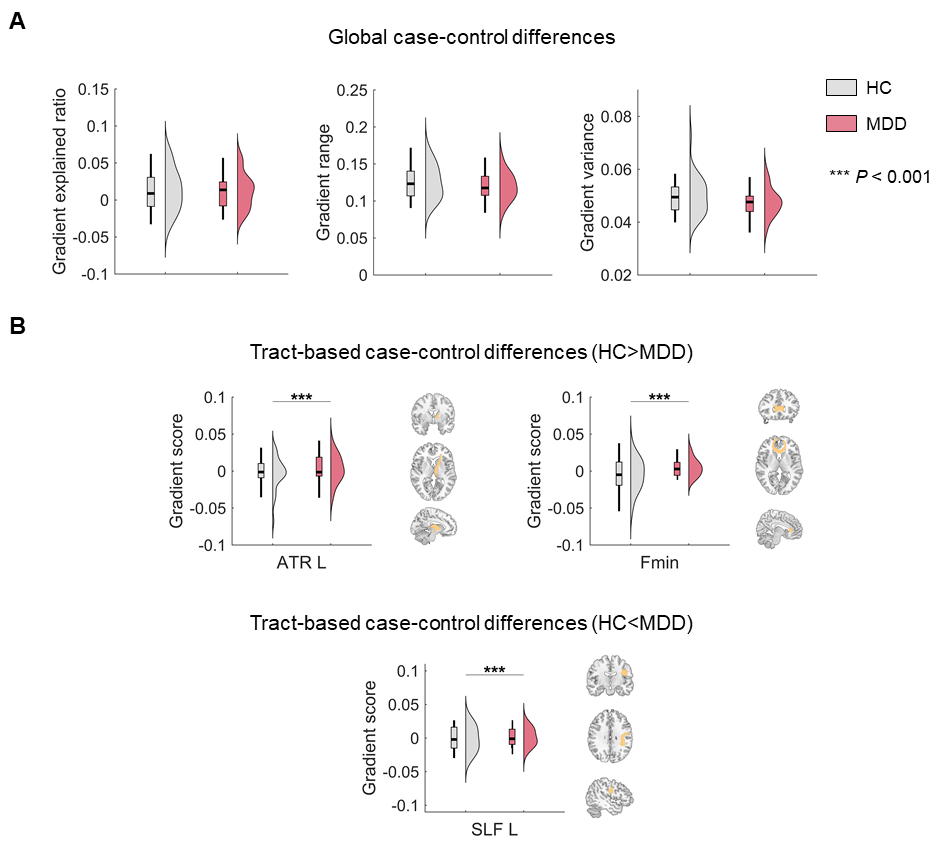
**

**Supplementary Figure 13.** Statistical comparison of the metrics of gradient 3 in HKH. (A) Case-control differences in the global gradient metrics of the second gradient. (B) Case-control differences in the tract-specific gradient scores of the second gradient. HKH, Hiroshima Kajikawa Hospital; ATR, anterior thalamic radiation; Fmin, forceps minor; SLF, superior longitudinal fasciculus; L, left; R, right. *** *P* < 0.001.

**
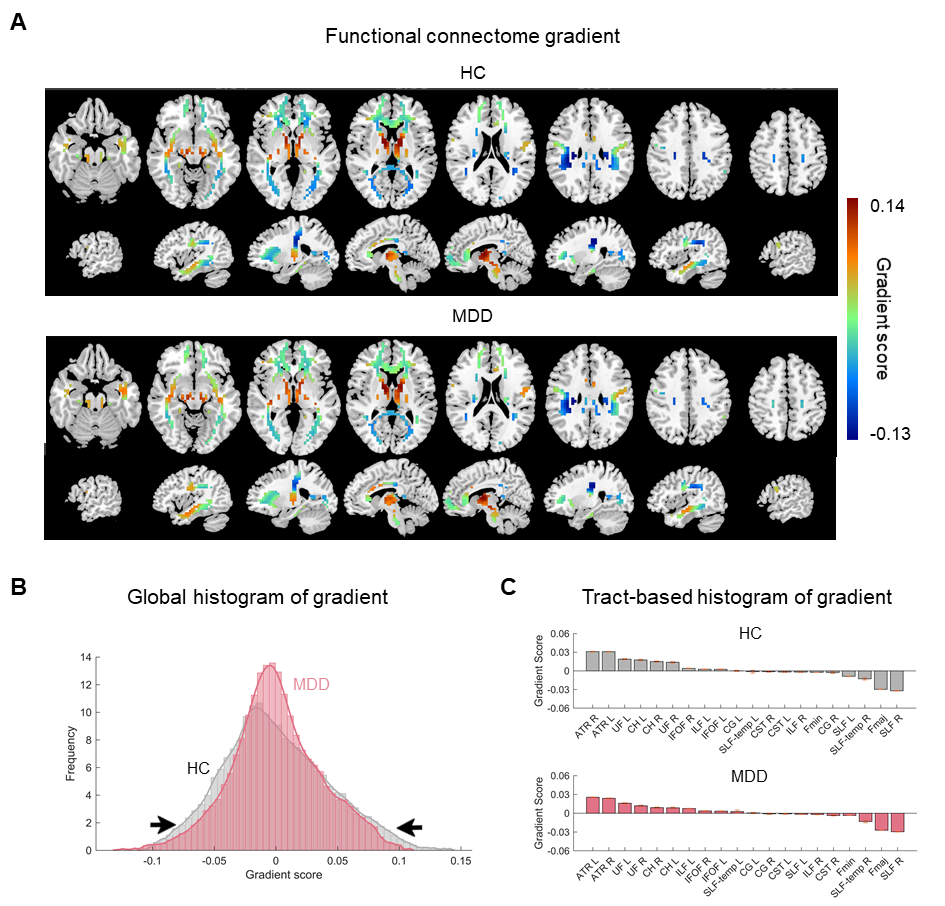
**

**Supplementary Figure 14.** WM functional gradient mapping in HC and patients with MDD under stricter motion control (mean FD < 0.2 mm). (A) In both HC and MDD groups, the principal WM functional gradient was organized along a continuous axis extending from the forceps major and superior longitudinal fasciculus to anterior thalamic radiation, exhibiting a superficial-to-deep WM pattern. (B) Global and (C) tract-based histograms showing that the extreme values were contracted in patients with MDD relative to HC. WM, white matter; HC, healthy controls; MDD, major depressive disorder; FD, framewise displacement; ATR, anterior thalamic radiation; UF, uncinate fasciculus; CST, corticospinal tract; IFOF, inferior fronto-occipital fasciculus; CG, cingulum (cingulate gyrus); CH, cingulum (hippocampus); ILF, inferior longitudinal fasciculus; SLF, superior longitudinal fasciculus; Fmin, forceps minor; Fmaj, forceps major; L, left; R, right.

**
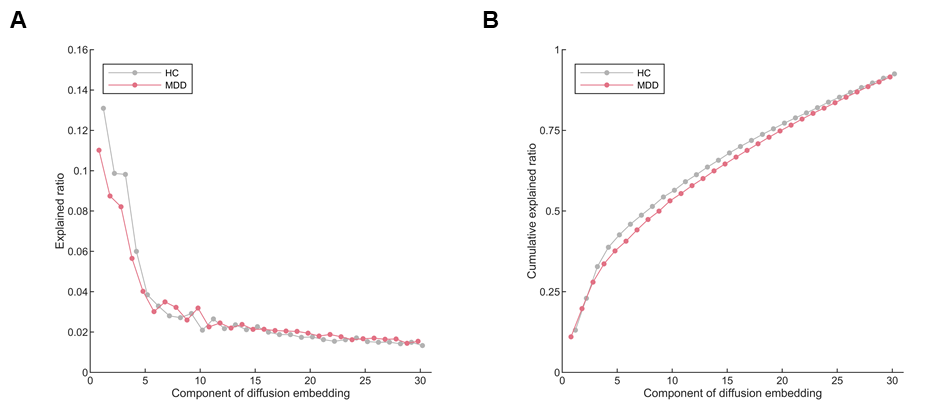
**

**Supplementary Figure 15.** The explained ratio of the gradients in white matter functional connectome under stricter motion control (mean FD < 0.2 mm). (A) The averaged explained ratio and (B**)** the cumulative averaged explained ratio of the first 30 diffusion embedding components in the HC and MDD groups. The HC group is shown in gray, and the MDD group is shown in red. The first three gradients explained 12.1% ± 3.8% of the total variance in the connectome across all individuals (MDD, 11.0% ± 3.5%; HC, 13.1% ± 4.1%). HC, healthy controls; MDD, major depressive disorder.

**
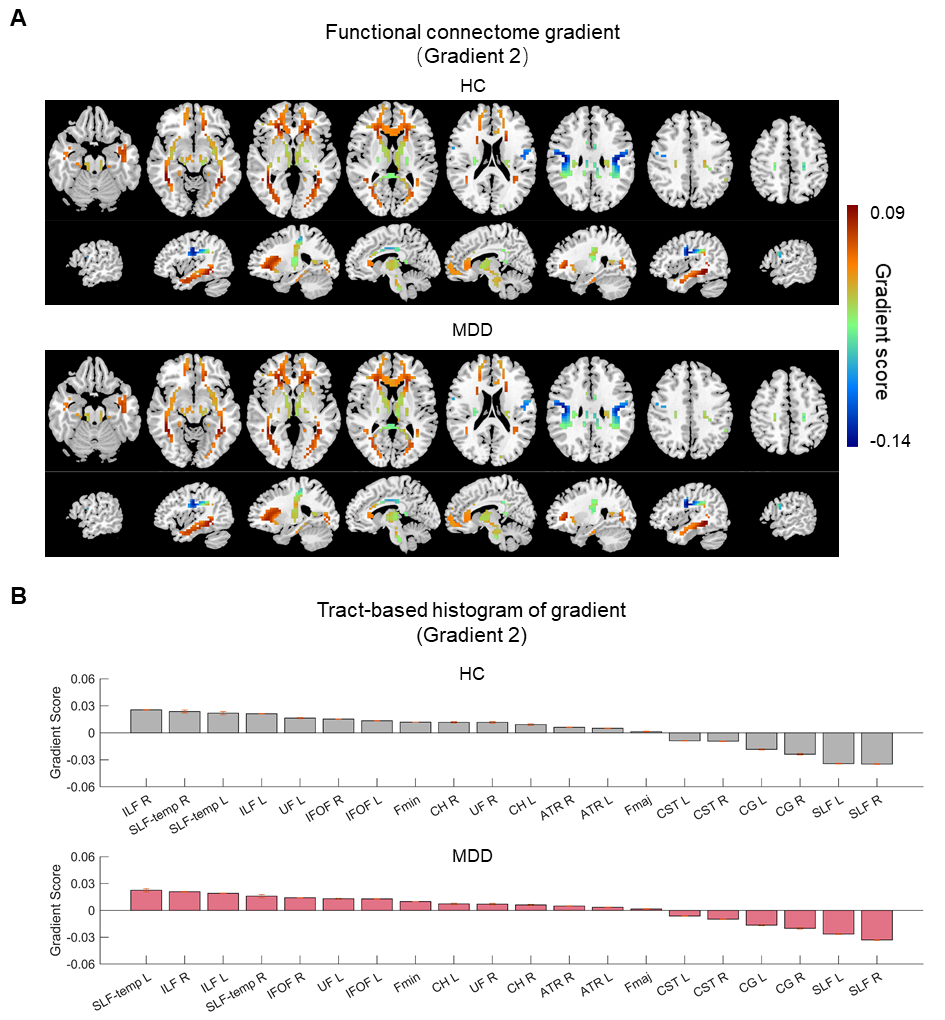
**

**Supplementary Figure 16.** WM functional gradient 2 in HC and patients with MDD under stricter motion control (mean FD < 0.2 mm). (A) In both HC and MDD groups, the second WM functional gradient extended between the inferior longitudinal fasciculus and the superior longitudinal fasciculus tracts. (B) Tract-based histograms showing that the extreme values were contracted in patients with MDD relative to HC. WM, white matter; HC, healthy controls; MDD, major depressive disorder; ATR, anterior thalamic radiation; CST, corticospinal tract; CG, cingulum (cingulate gyrus); CH, cingulum (hippocampus); Fmaj, forceps major; Fmin, forceps minor; IFOF, inferior fronto-occipital fasciculus; ILF, inferior longitudinal fasciculus; SLF, superior longitudinal fasciculus; UF, uncinate fasciculus; L, left; R, right.

**
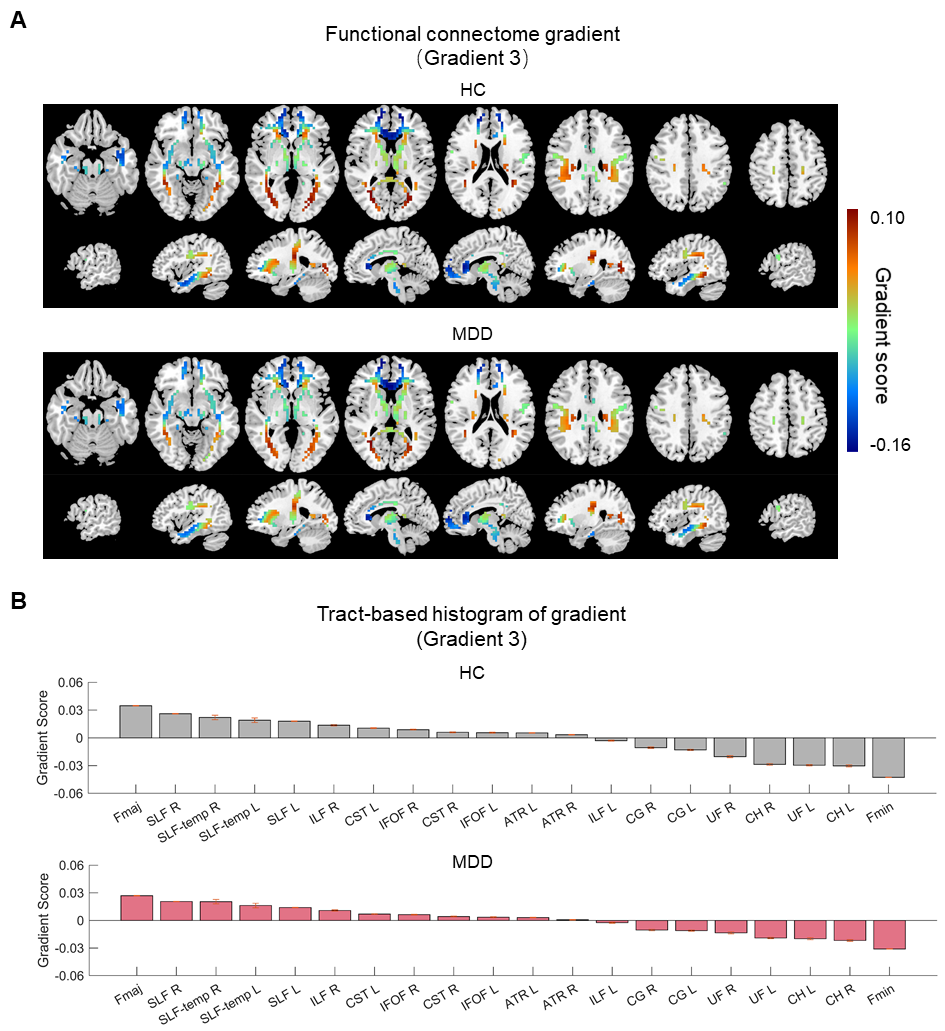
**

**Supplementary Figure 17.** WM functional gradient 3 in HC and patients with MDD under stricter motion control (mean FD < 0.2 mm). (A) In both HC and MDD groups, the third WM functional gradient extended between the forceps minor and the forceps major tracts. (B) Tract-based histograms showing that the extreme values were contracted in patients with MDD relative to HC. WM, white matter; HC, healthy controls; MDD, major depressive disorder; ATR, anterior thalamic radiation; CST, corticospinal tract; CG, cingulum (cingulate gyrus); CH, cingulum (hippocampus); Fmaj, forceps major; Fmin, forceps minor; IFOF, inferior fronto-occipital fasciculus; ILF, inferior longitudinal fasciculus; SLF, superior longitudinal fasciculus; UF, uncinate fasciculus; L, left; R, right.

**
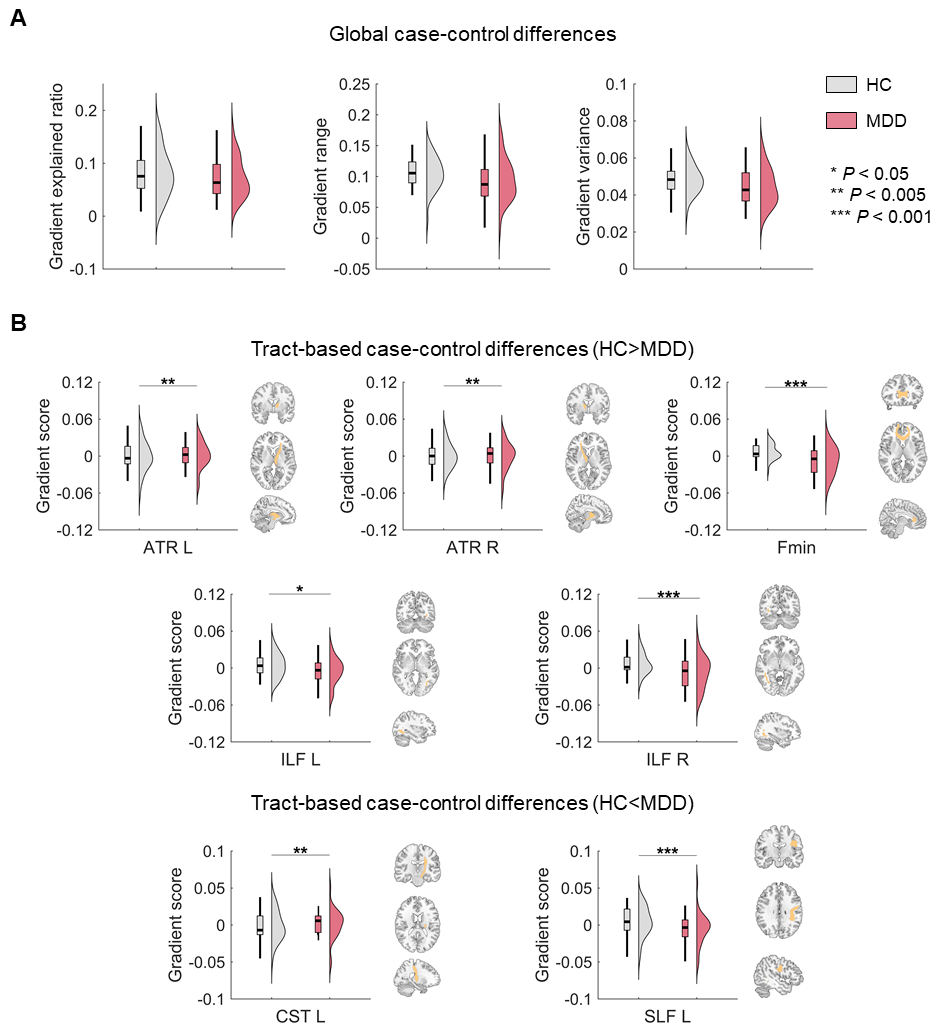
**

**Supplementary Figure 18.** Statistical comparison of the metrics of gradient 2 in participants with stricter motion control (mean FD < 0.2 mm). (A) Case-control differences in the global gradient metrics of the second gradient. (B) Case-control differences in the tract-specific gradient scores of the second gradient. SLF, superior longitudinal fasciculus; ILF, inferior longitudinal fasciculus; ATR, anterior thalamic radiation; CST, corticospinal tract; Fmin, forceps minor; L, left; R, right. * *P* < 0.05, ** *P* < 0.005, *** *P* < 0.001.

**
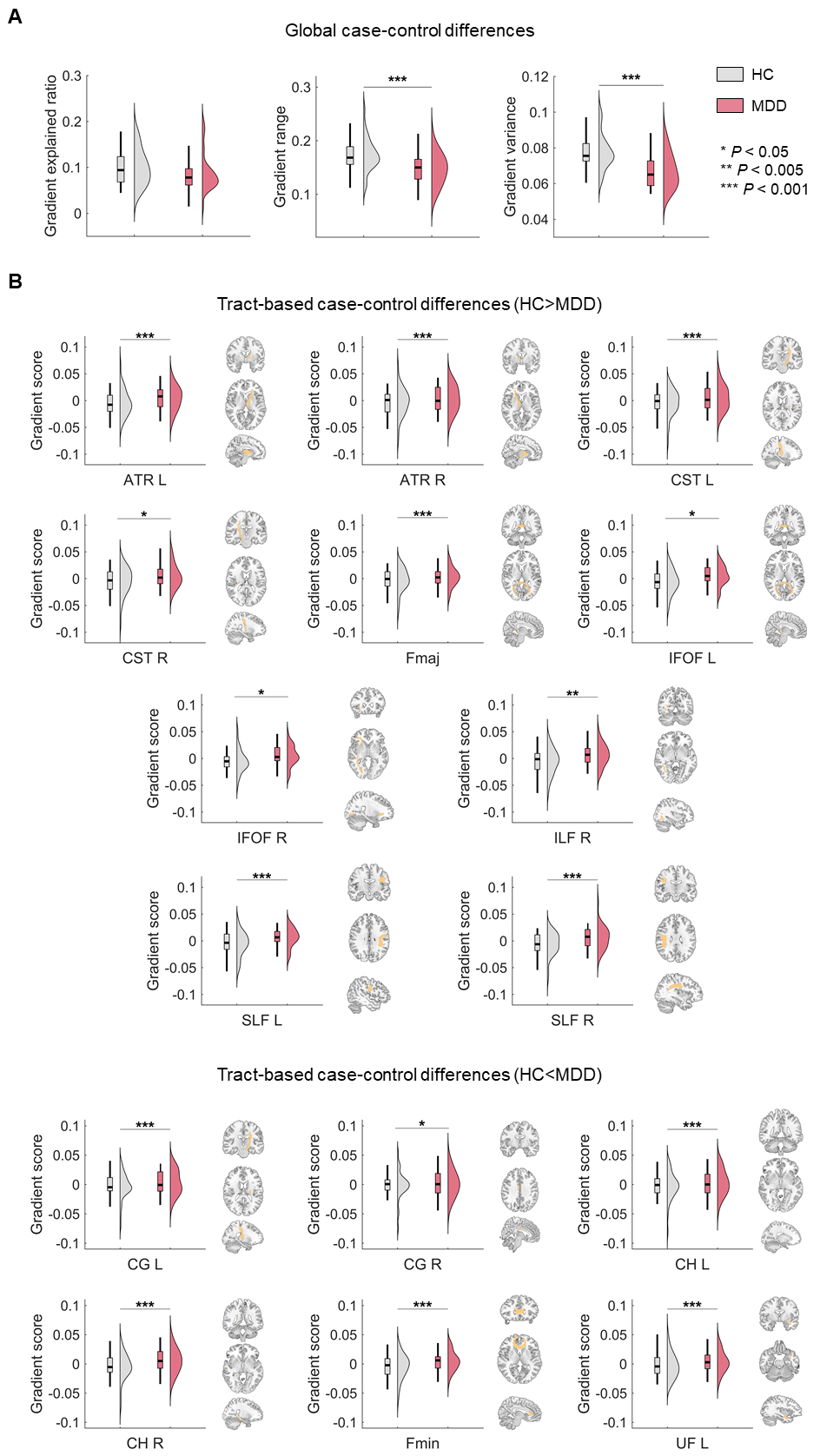
**

**Supplementary Figure 19.** Statistical comparison of the metrics of gradient 3 in participants with stricter motion control (mean FD < 0.2 mm). (A) Case-control differences in the global gradient metrics of the second gradient. (B) Case-control differences in the tract-specific gradient scores of the second gradient. ATR, anterior thalamic radiation; UF, uncinate fasciculus; CST, corticospinal tract; IFOF, inferior fronto-occipital fasciculus; CG, cingulum (cingulate gyrus); CH, cingulum (hippocampus); ILF, inferior longitudinal fasciculus; SLF, superior longitudinal fasciculus; Fmin, forceps minor; Fmaj, forceps major; L, left; R, right. * *P* < 0.05, ** *P* < 0.005, *** *P* < 0.001.

**
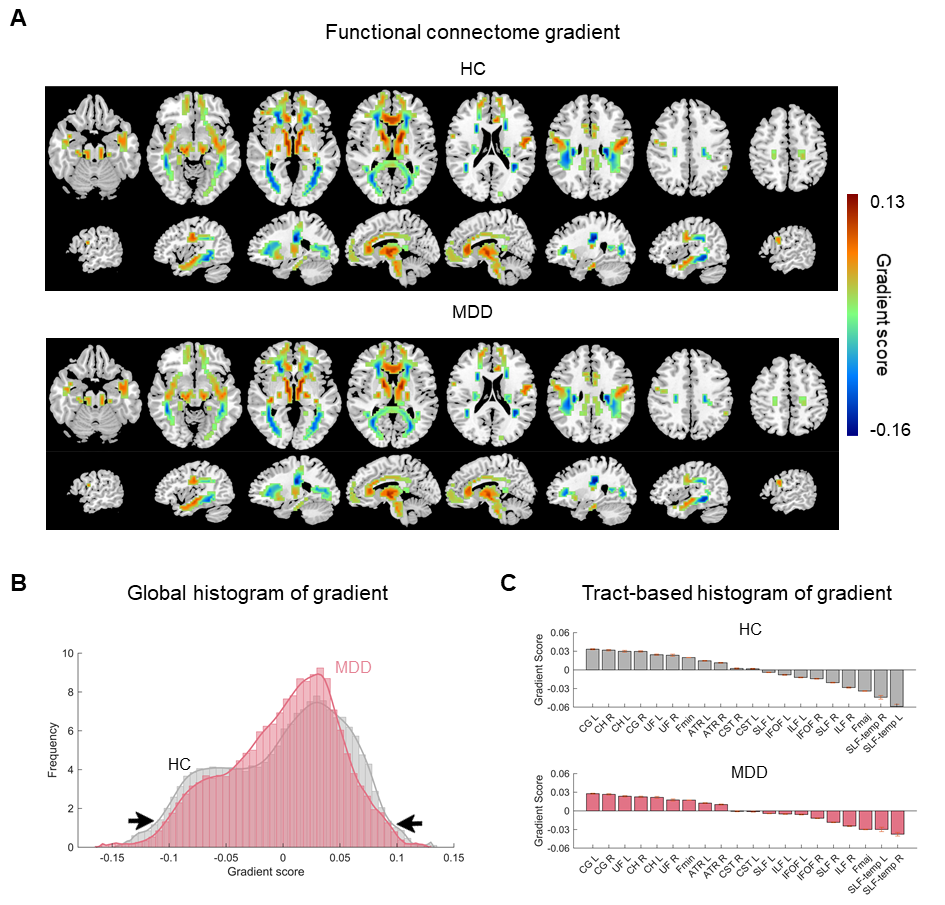
**

**Supplementary Figure 20.** WM functional gradient mapping in HC and patients with MDD in HKH. (A) In both HC and MDD groups, the principal WM functional gradient was organized along a continuous axis extending from the forceps major and superior longitudinal fasciculus to cingulate gyrus. (B) Global and (C) tract-based histograms showing that the extreme values were contracted in patients with MDD relative to HC. WM, white matter; HC, healthy controls; MDD, major depressive disorder; HKH, Hiroshima Kajikawa Hospital; ATR, anterior thalamic radiation; UF, uncinate fasciculus; CST, corticospinal tract; IFOF, inferior fronto-occipital fasciculus; CG, cingulum (cingulate gyrus); CH, cingulum (hippocampus); ILF, inferior longitudinal fasciculus; SLF, superior longitudinal fasciculus; Fmin, forceps minor; Fmaj, forceps major; L, left; R, right.

**
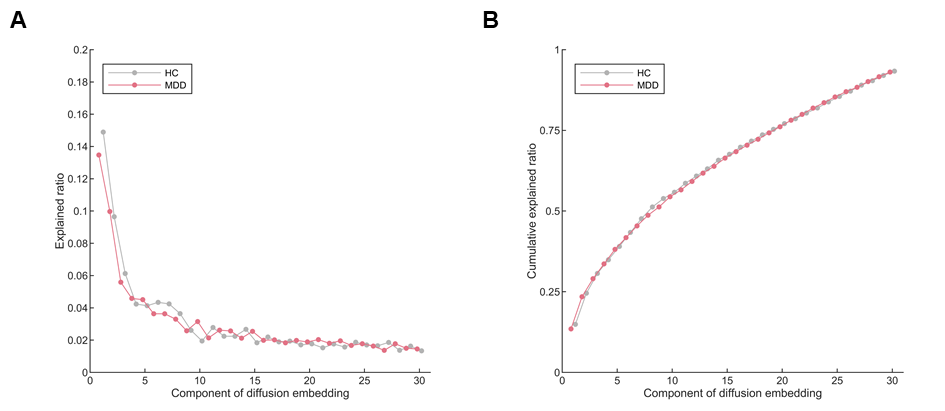
**

**Supplementary Figure 21.** The explained ratio of the gradients in white matter functional connectome in HKH. (A) The averaged explained ratio and (B**)** the cumulative averaged explained ratio of the first 30 diffusion embedding components in the HC and MDD groups. The HC group is shown in gray, and the MDD group is shown in red. The first three gradients explained 14.2% ± 5.0% of the total variance in the connectome across all individuals (MDD, 13.5% ± 4.7%; HC, 14.9% ± 5.3%). HC, healthy controls; MDD, major depressive disorder; HKH, Hiroshima Kajikawa Hospital.

**
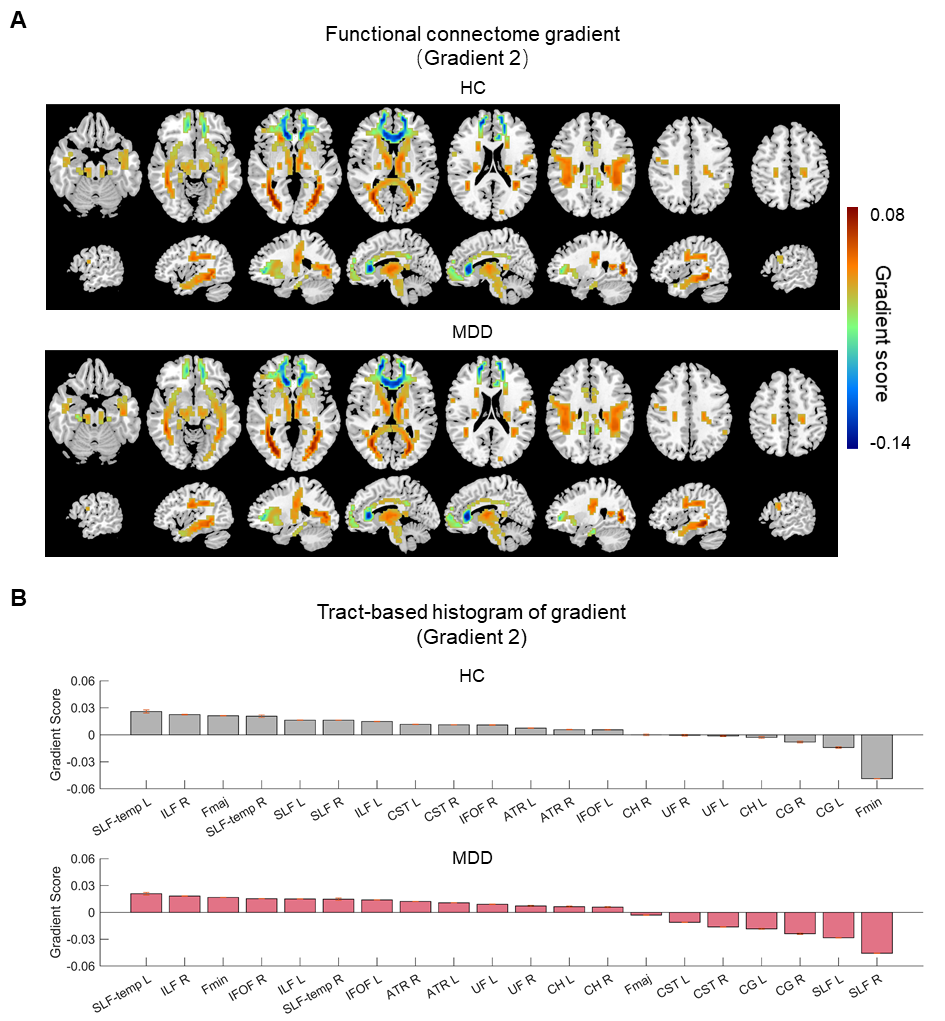
**

**Supplementary Figure 22.** WM functional gradient 2 in HC and patients with MDD in HKH. (A) In both HC and MDD groups, the second WM functional gradient extended between the forceps minor and the superior longitudinal fasciculus tracts. (B) Tract-based histograms showing that the extreme values were contracted in patients with MDD relative to HC. WM, white matter; HC, healthy controls; MDD, major depressive disorder; HKH, Hiroshima Kajikawa Hospital; ATR, anterior thalamic radiation; CST, corticospinal tract; CG, cingulum (cingulate gyrus); CH, cingulum (hippocampus); Fmaj, forceps major; Fmin, forceps minor; IFOF, inferior fronto-occipital fasciculus; ILF, inferior longitudinal fasciculus; SLF, superior longitudinal fasciculus; UF, uncinate fasciculus; L, left; R, right.

**
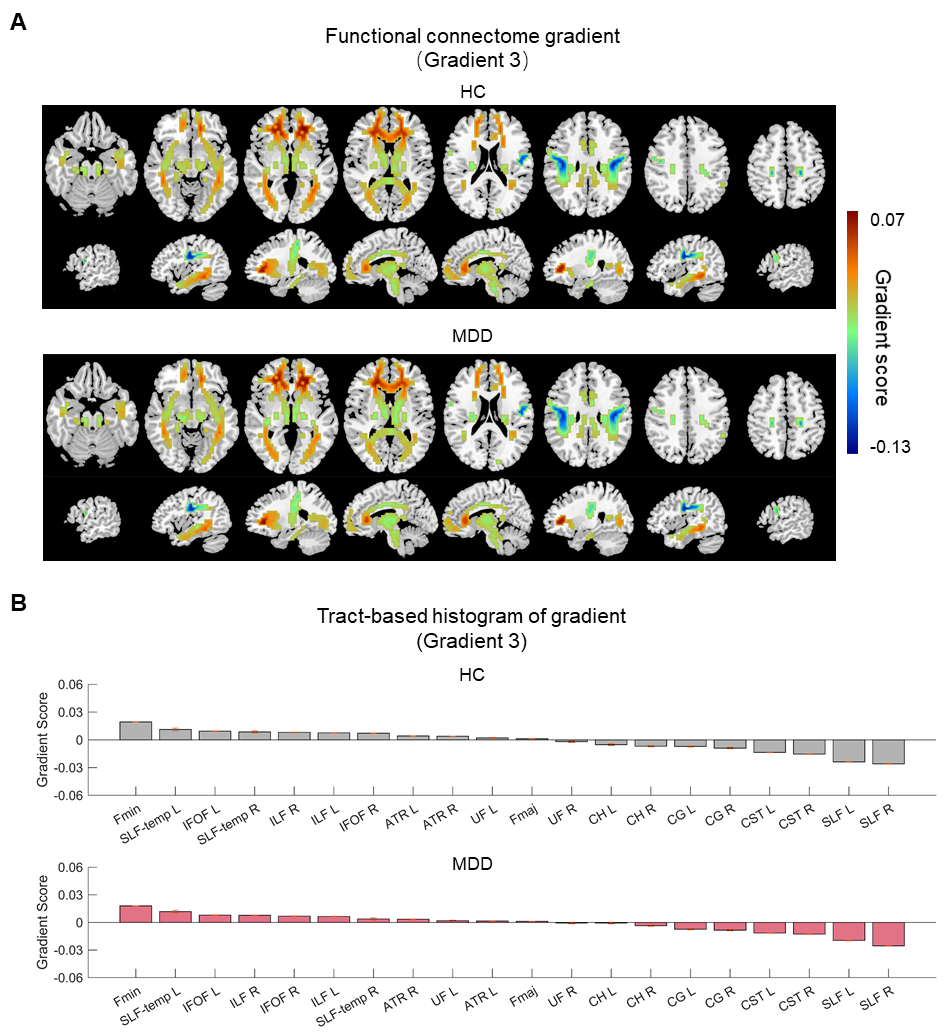
**

**Supplementary Figure 23.** WM functional gradient 3 in HC and patients with MDD in HKH. (A) In both HC and MDD groups, the second WM functional gradient extended between the forceps minor and the superior longitudinal fasciculus tracts. (B) Tract-based histograms showing that the extreme values were contracted in patients with MDD relative to HC. WM, white matter; HC, healthy controls; MDD, major depressive disorder; HKH, Hiroshima Kajikawa Hospital; ATR, anterior thalamic radiation; CST, corticospinal tract; CG, cingulum (cingulate gyrus); CH, cingulum (hippocampus); Fmaj, forceps major; Fmin, forceps minor; IFOF, inferior fronto-occipital fasciculus; ILF, inferior longitudinal fasciculus; SLF, superior longitudinal fasciculus; UF, uncinate fasciculus; L, left; R, right.

**Supplementary Tables**

**Supplementary Table 1. White matter tracts from JHU DTI-based WM atlas**

| Number | Tracts | Full name |
| --- | --- | --- |
| 1 | ATR L | anterior thalamic radiation left |
| 2 | ATR R | anterior thalamic radiation right |
| 3 | CST L | corticospinal tract left |
| 4 | CST R | corticospinal tract right |
| 5 | CG L | cingulum (cingulate gyrus) left |
| 6 | CG R | cingulum (cingulate gyrus) right |
| 7 | CH L | cingulum (hippocampus) left |
| 8 | CH R | cingulum (hippocampus) right |
| 9 | Fmaj | forceps major |
| 10 | Fmin | forceps minor |
| 11 | IFOF L | inferior fronto-occipital fasciculus left |
| 12 | IFOF R | inferior fronto-occipital fasciculus right |
| 13 | ILF L | inferior longitudinal fasciculus left |
| 14 | ILF R | inferior longitudinal fasciculus right |
| 15 | SLF L | superior longitudinal fasciculus left |
| 16 | SLF R | superior longitudinal fasciculus right |
| 17 | UF L | uncinate fasciculus left |
| 18 | UF R | uncinate fasciculus right |
| 19 | SLF-temp L | superior longitudinal fasciculus temp left |
| 20 | SLF-temp R | superior longitudinal fasciculus temp right |

**Supplementary Table 2. Demographic and clinical characteristics of participants in HKH**

|  | MDD (n=29) | HC (n=27) | *t* or *χ2*/*P* |
| --- | --- | --- | --- |
| Age, mean (SD), yr | 45.72(11.84) | 45.89(8.73) | 0.19/0.85 |
| Sex (M/F) | 18/11 | 10/17 | 3.51/0.06 |
| BDI-II, mean (SD) | 27.52(8.67) | 5.44(4.59) | 11.77/<0.001 |
| Mean FD, mean (SD), mm | 0.12(0.04) | 0.12(0.05) | -0.43/0.67 |
| Maximum Translation,  mean (SD), mm | 0.48(0.24) | 0.51(0.34) | -0.47/0.64 |
| Maximum Rotation,  mean (SD), degree | 0.59(0.41) | 0.52(0.35) | 0.65/0.52 |

Abbreviations: MDD, major depressive disorder; HC, healthy controls; SD, standard deviation; M, male; F, female; BDI-II, Beck Depression Inventory-Second Edition; FD, framewise displacement.

**Supplementary Table 3. Case-control differences in the global gradient metrics**

| Metric | MDD (N=48),  mean (SD) | HC (N=68),  mean (SD) | *t* | Cohen’s *d* | *P* | FDR *q* |
| --- | --- | --- | --- | --- | --- | --- |
| G1 explained ratio | 0.118 (0.038) | 0.133 (0.034) | -2.16 | -0.41 | 0.0330 | 0.0989 |
| G1 range | 0.171 (0.038) | 0.194 (0.031) | -3.48 | -0.66 | 0.0007 | 0.0012 |
| G1 variance | 0.043 (0.014) | 0.051 (0.012) | -3.45 | -0.65 | 0.0008 | 0.0012 |
| G2 explained ratio | 0.075 (0.037) | 0.081 (0.039) | -0.79 | -0.15 | 0.4314 | 0.4314 |
| G2 range | 0.123 (0.033) | 0.129 (0.025) | -1.29 | -0.24 | 0.2014 | 0.3560 |
| G2 variance | 0.024 (0.010) | 0.026 (0.008) | -1.19 | -0.22 | 0.2373 | 0.3560 |
| G3 explained ratio | 0.060 (0.022) | 0.062 (0.025) | -0.51 | -0.10 | 0.6139 | 0.6139 |
| G3 range | 0.110 (0.019) | 0.119 (0.025) | -2.23 | -0.42 | 0.0277 | 0.0416 |
| G3 variance | 0.022 (0.006) | 0.025 (0.007) | -2.57 | -0.49 | 0.0114 | 0.0341 |

Abbreviations: MDD, major depressive disorder; HC, healthy controls; SD, standard deviation; G1, Gradient 1; G2, Gradient 2; G3, Gradient 3.

**Supplementary Table 4. Case-control differences in the gradient scores of the tracts in G1**

| Tracts | *t* | Cohen’s *d* | *P* | FDR *q* |
| --- | --- | --- | --- | --- |
| ATR L | -3.10 | -0.27 | 0.002 | 0.004 |
| ATR R | -1.96 | -0.18 | 0.053 | 0.074 |
| CST L | -0.43 | -0.04 | 0.671 | 0.671 |
| CST R | -1.94 | -0.22 | 0.056 | 0.074 |
| CG L | -7.07 | -1.31 | <1.0×10^-16^ | <1.0×10^-16^ |
| CG R | -4.73 | -1.18 | <1.0×10^-16^ | 0.001 |
| CH L | -6.67 | -2.36 | <1.0×10^-16^ | 0.001 |
| CH R | -4.72 | -1.36 | 0.001 | 0.001 |
| Fmaj | 11.33 | 1.14 | <1.0×10^-16^ | <1.0×10^-16^ |
| Fmin | -15.83 | -0.93 | <1.0×10^-16^ | <1.0×10^-16^ |
| IFOF L | 0.80 | 0.09 | 0.425 | 0.447 |
| IFOF R | 2.00 | 0.19 | 0.048 | 0.074 |
| ILF L | 4.12 | 0.42 | <1.0×10^-16^ | <1.0×10^-16^ |
| ILF R | 5.23 | 0.70 | <1.0×10^-16^ | <1.0×10^-16^ |
| SLF L | 3.57 | 0.29 | <1.0×10^-16^ | 0.001 |
| SLF R | 5.21 | 0.46 | <1.0×10^-16^ | <1.0×10^-16^ |
| UF L | -3.78 | -0.82 | 0.001 | 0.002 |
| UF R | -0.93 | -0.29 | 0.377 | 0.419 |
| SLF-temp L | 4.96 | 3.51 | 0.127 | 0.158 |
| SLF-temp R | 2.14 | 1.24 | 0.166 | 0.195 |

Abbreviations: ATR, anterior thalamic radiation; CST, corticospinal tract; CG, cingulum (cingulate gyrus); CH, cingulum (hippocampus); Fmaj, forceps major; Fmin, forceps minor; IFOF, inferior fronto-occipital fasciculus; ILF, inferior longitudinal fasciculus; SLF, superior longitudinal fasciculus; UF, uncinate fasciculus; L, left; R, right.

**Supplementary Table 5. Case-control differences in the gradient scores of the tracts in G2**

| Tracts | *t* | Cohen’s *d* | *P* | FDR *q* |
| --- | --- | --- | --- | --- |
| ATR L | -8.22 | -0.71 | <1.0×10^-16^ | <1.0×10^-16^ |
| ATR R | -8.85 | -0.83 | <1.0×10^-16^ | <1.0×10^-16^ |
| CST L | 1.39 | 0.14 | 0.168 | 0.247 |
| CST R | -1.32 | -0.15 | 0.191 | 0.247 |
| CG L | 2.29 | 0.43 | 0.030 | 0.059 |
| CG R | 1.76 | 0.44 | 0.098 | 0.178 |
| CH L | -0.44 | -0.15 | 0.676 | 0.675 |
| CH R | -1.29 | -0.37 | 0.223 | 0.248 |
| Fmaj | 1.32 | 0.13 | 0.189 | 0.248 |
| Fmin | -5.57 | -0.33 | <1.0×10^-16^ | <1.0×10^-16^ |
| IFOF L | -1.58 | -0.17 | 0.119 | 0.198 |
| IFOF R | -2.56 | -0.24 | 0.012 | 0.030 |
| ILF L | -1.26 | -0.13 | 0.212 | 0.248 |
| ILF R | -11.91 | -1.59 | <1.0×10^-16^ | <1.0×10^-16^ |
| SLF L | 17.24 | 1.40 | <1.0×10^-16^ | <1.0×10^-16^ |
| SLF R | 4.28 | 0.38 | <1.0×10^-16^ | <1.0×10^-16^ |
| UF L | -3.66 | -0.80 | 0.002 | 0.004 |
| UF R | -0.77 | -0.24 | 0.460 | 0.484 |
| SLF-temp L | -2.81 | -1.99 | 0.217 | 0.248 |
| SLF-temp R | -7.21 | -4.16 | 0.019 | 0.041 |

Abbreviations: ATR, anterior thalamic radiation; CST, corticospinal tract; CG, cingulum (cingulate gyrus); CH, cingulum (hippocampus); Fmaj, forceps major; Fmin, forceps minor; IFOF, inferior fronto-occipital fasciculus; ILF, inferior longitudinal fasciculus; SLF, superior longitudinal fasciculus; UF, uncinate fasciculus; L, left; R, right.

**Supplementary Table 6. Case-control differences in the gradient scores of the tracts in G3**

| Tracts | *t* | Cohen’s *d* | *P* | FDR *q* |
| --- | --- | --- | --- | --- |
| ATR L | -9.90 | -0.85 | <1.0×10^-16^ | <1.0×10^-16^ |
| ATR R | -6.98 | -0.65 | <1.0×10^-16^ | <1.0×10^-16^ |
| CST L | -5.99 | -0.61 | <1.0×10^-16^ | <1.0×10^-16^ |
| CST R | -6.80 | -0.78 | <1.0×10^-16^ | <1.0×10^-16^ |
| CG L | -1.72 | -0.32 | 0.096 | 0.120 |
| CG R | 1.01 | 0.25 | 0.331 | 0.367 |
| CH L | 4.93 | 1.74 | 0.002 | 0.003 |
| CH R | -0.32 | -0.09 | 0.753 | 0.753 |
| Fmaj | -20.29 | -2.04 | <1.0×10^-16^ | <1.0×10^-16^ |
| Fmin | 31.55 | 1.85 | <1.0×10^-16^ | <1.0×10^-16^ |
| IFOF L | -4.53 | -0.49 | <1.0×10^-16^ | <1.0×10^-16^ |
| IFOF R | -4.27 | -0.41 | <1.0×10^-16^ | <1.0×10^-16^ |
| ILF L | 1.84 | 0.19 | 0.068 | 0.098 |
| ILF R | -2.86 | -0.38 | 0.006 | 0.010 |
| SLF L | 1.68 | 0.14 | 0.095 | 0.120 |
| SLF R | -7.94 | -0.71 | <1.0×10^-16^ | <1.0×10^-16^ |
| UF L | 6.46 | 1.41 | <1.0×10^-16^ | <1.0×10^-16^ |
| UF R | 2.58 | 0.82 | 0.030 | 0.046 |
| SLF-temp L | 4.89 | 3.46 | 0.128 | 0.151 |
| SLF-temp R | -0.90 | -0.52 | 0.462 | 0.486 |

Abbreviations: ATR, anterior thalamic radiation; CST, corticospinal tract; CG, cingulum (cingulate gyrus); CH, cingulum (hippocampus); Fmaj, forceps major; Fmin, forceps minor; IFOF, inferior fronto-occipital fasciculus; ILF, inferior longitudinal fasciculus; SLF, superior longitudinal fasciculus; UF, uncinate fasciculus; L, left; R, right.

**Supplementary Table 7. Correlation between mean FD and global gradient metrics**

| Metric | *r* | *P* | FDR *q* |
| --- | --- | --- | --- |
| G1 explained ratio | <1.0×10^-16^ | 1 | 1 |
| G1 range | <1.0×10^-16^ | 1 | 1 |
| G1 variance | <1.0×10^-16^ | 1 | 1 |
| G2 explained ratio | <1.0×10^-16^ | 1 | 1 |
| G2 range | <1.0×10^-16^ | 1 | 1 |
| G2 variance | <1.0×10^-16^ | 1 | 1 |
| G3 explained ratio | <1.0×10^-16^ | 1 | 1 |
| G3 range | <1.0×10^-16^ | 1 | 1 |
| G3 variance | <1.0×10^-16^ | 1 | 1 |

Abbreviations: FD, framewise displacement; G1, Gradient 1; G2, Gradient 2; G3, Gradient 3.

**Supplementary Table 8. Demographic and clinical characteristics of participants under stricter motion control (mean FD < 0.2 mm)**

|  | MDD (n=30) | HC (n=33) | t or χ^2^/*P* |
| --- | --- | --- | --- |
| Age, mean (SD), yr | 43.07(10.71) | 47.36(10.23) | -1.63/0.11 |
| Sex (M/F) | 17/13 | 9/24 | 5.60/0.02 |
| BDI-II, mean (SD) | 25.00(8.57) | 8.58(6.45) | 8.65/<0.001 |
| Mean FD, mean (SD), mm | 0.15(0.03) | 0.15(0.03) | -0.54/0.59 |
| Maximum Translation,  mean (SD), mm | 1.23(0.72) | 1.09(0.54) | 0.86/0.39 |
| Maximum Rotation,  mean (SD), degree | 0.80(0.47) | 0.86(0.40) | -0.49/0.63 |

Abbreviations: FD, framewise displacement; MDD, major depressive disorder; HC, healthy controls; SD, standard deviation; M, male; F, female; BDI-II, Beck Depression Inventory-Second Edition.

**Supplementary Table 9. Spatial Correlations of gradients maps between cohorts with 0.3 mm and 0.2 mm mean FD thresholds**

| Gradient map | *r* | *P* | FDR *q* |
| --- | --- | --- | --- |
| G1 MDD | 0.7997 | <1.0×10^-16^ | <1.0×10^-16^ |
| G1 HC | 0.7997 | <1.0×10^-16^ | <1.0×10^-16^ |
| G2 MDD | 0.7572 | <1.0×10^-16^ | <1.0×10^-16^ |
| G2 HC | 0.7572 | <1.0×10^-16^ | <1.0×10^-16^ |
| G3 MDD | 0.8038 | <1.0×10^-16^ | <1.0×10^-16^ |
| G3 HC | 0.8038 | <1.0×10^-16^ | <1.0×10^-16^ |

Abbreviations: MDD, major depressive disorder; HC, healthy controls; SD, standard deviation; G1, Gradient 1; G2, Gradient 2; G3, Gradient 3.

**Supplementary Table 10. Case-control differences in the gradient scores of the tracts in G1** **under stricter motion control (mean FD < 0.2 mm)**

| Tracts | *t* | Cohen’s *d* | *P* | FDR *q* |
| --- | --- | --- | --- | --- |
| ATR L | -5.26 | -0.45 | <1.0×10^-16^ | <1.0×10^-16^ |
| ATR R | -7.48 | -0.70 | <1.0×10^-16^ | <1.0×10^-16^ |
| CST L | -0.21 | -0.02 | 0.838 | 0.838 |
| CST R | -2.74 | -0.31 | 0.008 | 0.019 |
| CG L | -1.36 | -0.25 | 0.185 | 0.336 |
| CG R | -1.17 | -0.29 | 0.260 | 0.401 |
| CH L | -3.30 | -1.17 | 0.013 | 0.029 |
| CH R | -3.43 | -0.99 | 0.006 | 0.016 |
| Fmaj | 5.83 | 0.59 | <1.0×10^-16^ | <1.0×10^-16^ |
| Fmin | -3.28 | -0.19 | 0.001 | 0.004 |
| IFOF L | 0.74 | 0.08 | 0.461 | 0.614 |
| IFOF R | -0.29 | -0.03 | 0.776 | 0.838 |
| ILF L | 7.97 | 0.81 | <1.0×10^-16^ | <1.0×10^-16^ |
| ILF R | 0.84 | 0.11 | 0.407 | 0.581 |
| SLF L | 14.26 | 1.16 | <1.0×10^-16^ | <1.0×10^-16^ |
| SLF R | 0.59 | 0.05 | 0.556 | 0.696 |
| UF L | -1.27 | -0.28 | 0.219 | 0.364 |
| UF R | -0.31 | -0.10 | 0.762 | 0.838 |
| SLF-temp L | 8.67 | 6.13 | 0.073 | 0.146 |
| SLF-temp R | -0.26 | -0.15 | 0.817 | 0.838 |

Abbreviations: FD, framewise displacement; ATR, anterior thalamic radiation; CST, corticospinal tract; CG, cingulum (cingulate gyrus); CH, cingulum (hippocampus); Fmaj, forceps major; Fmin, forceps minor; IFOF, inferior fronto-occipital fasciculus; ILF, inferior longitudinal fasciculus; SLF, superior longitudinal fasciculus; UF, uncinate fasciculus; L, left; R, right.

**Supplementary Table 11. Correlation between gradients maps of COI and HKH cohorts**

| Groups | *r* | *P* | FDR *q* |
| --- | --- | --- | --- |
| G1 MDD | 0.6237 | <1.0×10^-16^ | <1.0×10^-16^ |
| G1 HC | 0.6237 | <1.0×10^-16^ | <1.0×10^-16^ |
| G2 MDD | 0.1463 | <1.0×10^-16^ | <1.0×10^-16^ |
| G2 HC | 0.1463 | <1.0×10^-16^ | <1.0×10^-16^ |
| G3 MDD | 0.0384 | 0.1321 | 0.1321 |
| G3 HC | 0.0384 | 0.1321 | 0.1321 |

Abbreviations: MDD, major depressive disorder; HC, healthy controls; SD, standard deviation; G1, Gradient 1; G2, Gradient 2; G3, Gradient 3.

**Supplementary Table 12. Case-control differences in G1 scores of the tracts in HKH**

| Tracts | *t* | Cohen’s *d* | P | FDR *q* |
| --- | --- | --- | --- | --- |
| ATR L | -1.65 | -0.14 | 0.101 | 0.144 |
| ATR R | 0.03 | 0.00 | 0.975 | 0.975 |
| CST L | -3.03 | -0.31 | 0.003 | 0.007 |
| CST R | -2.73 | -0.31 | 0.008 | 0.016 |
| CG L | -6.02 | -1.12 | <1.0×10^-16^ | <1.0×10^-16^ |
| CG R | -3.84 | -0.96 | 0.002 | 0.005 |
| CH L | -4.49 | -1.59 | 0.003 | 0.007 |
| CH R | -6.31 | -1.82 | <1.0×10^-16^ | <1.0×10^-16^ |
| Fmaj | 3.82 | 0.38 | <1.0×10^-16^ | 0.001 |
| Fmin | -7.26 | -0.43 | <1.0×10^-16^ | <1.0×10^-16^ |
| IFOF L | 1.42 | 0.15 | 0.159 | 0.199 |
| IFOF R | 2.65 | 0.25 | 0.009 | 0.017 |
| ILF L | 9.20 | 0.93 | <1.0×10^-16^ | <1.0×10^-16^ |
| ILF R | 3.75 | 0.50 | <1.0×10^-16^ | 0.001 |
| SLF L | -0.94 | -0.08 | 0.347 | 0.385 |
| SLF R | 2.31 | 0.21 | 0.022 | 0.038 |
| UF L | -0.66 | -0.14 | 0.514 | 0.541 |
| UF R | -2.19 | -0.69 | 0.056 | 0.086 |
| SLF-temp L | 4.35 | 3.08 | 0.144 | 0.192 |
| SLF-temp R | 1.63 | 0.94 | 0.245 | 0.288 |

Abbreviations: ATR, anterior thalamic radiation; CST, corticospinal tract; CG, cingulum (cingulate gyrus); CH, cingulum (hippocampus); Fmaj, forceps major; Fmin, forceps minor; IFOF, inferior fronto-occipital fasciculus; ILF, inferior longitudinal fasciculus; SLF, superior longitudinal fasciculus; UF, uncinate fasciculus; L, left; R, right.

**Supplementary Table 13. Case-control differences in G2 scores of the tracts in HKH**

| Tracts | *t* | Cohen’s *d* | *P* | FDR *q* |
| --- | --- | --- | --- | --- |
| ATR L | -1.28 | -0.11 | 0.202 | 0.311 |
| ATR R | -1.92 | -0.18 | 0.057 | 0.104 |
| CST L | -1.49 | -0.15 | 0.139 | 0.232 |
| CST R | -0.19 | -0.02 | 0.851 | 0.896 |
| CG L | 7.50 | 1.39 | <1.0×10^-16^ | <1.0×10^-16^ |
| CG R | 2.09 | 0.52 | 0.054 | 0.104 |
| CH L | -1.06 | -0.37 | 0.326 | 0.404 |
| CH R | -2.35 | -0.68 | 0.039 | 0.086 |
| Fmaj | -3.62 | -0.36 | <1.0×10^-16^ | 0.002 |
| Fmin | 9.11 | 0.53 | <1.0×10^-16^ | <1.0×10^-16^ |
| IFOF L | -0.99 | -0.11 | 0.325 | 0.404 |
| IFOF R | -1.08 | -0.10 | 0.282 | 0.402 |
| ILF L | -3.42 | -0.35 | 0.001 | 0.004 |
| ILF R | -2.88 | -0.38 | 0.006 | 0.019 |
| SLF L | -3.76 | -0.31 | <1.0×10^-16^ | 0.002 |
| SLF R | -0.03 | 0.00 | 0.974 | 0.974 |
| UF L | -0.55 | -0.12 | 0.591 | 0.657 |
| UF R | -2.44 | -0.77 | 0.037 | 0.086 |
| SLF-temp L | -17.45 | -12.34 | 0.036 | 0.086 |
| SLF-temp R | -1.23 | -0.71 | 0.343 | 0.404 |

Abbreviations: ATR, anterior thalamic radiation; CST, corticospinal tract; CG, cingulum (cingulate gyrus); CH, cingulum (hippocampus); Fmaj, forceps major; Fmin, forceps minor; IFOF, inferior fronto-occipital fasciculus; ILF, inferior longitudinal fasciculus; SLF, superior longitudinal fasciculus; UF, uncinate fasciculus; L, left; R, right.

**Supplementary Table 14. Case-control differences in G3 scores of the tracts in HKH**

| Tracts | *t* | Cohen’s *d* | *P* | FDR *q* |
| --- | --- | --- | --- | --- |
| ATR L | -8.47 | -0.73 | <1.0×10^-16^ | <1.0×10^-16^ |
| ATR R | 1.38 | 0.13 | 0.171 | 0.264 |
| CST L | 2.50 | 0.26 | 0.014 | 0.069 |
| CST R | 1.55 | 0.18 | 0.126 | 0.251 |
| CG L | 0.02 | 0.00 | 0.986 | 0.986 |
| CG R | 0.19 | 0.05 | 0.851 | 0.933 |
| CH L | 3.11 | 1.10 | 0.017 | 0.069 |
| CH R | 2.27 | 0.66 | 0.044 | 0.126 |
| Fmaj | 1.75 | 0.18 | 0.083 | 0.184 |
| Fmin | -5.14 | -0.30 | <1.0×10^-16^ | <1.0×10^-16^ |
| IFOF L | -1.20 | -0.13 | 0.234 | 0.312 |
| IFOF R | 0.14 | 0.01 | 0.887 | 0.933 |
| ILF L | -0.96 | -0.10 | 0.339 | 0.399 |
| ILF R | -1.10 | -0.15 | 0.278 | 0.348 |
| SLF L | 6.21 | 0.51 | <1.0×10^-16^ | <1.0×10^-16^ |
| SLF R | -2.15 | -0.19 | 0.034 | 0.112 |
| UF L | 1.25 | 0.27 | 0.227 | 0.312 |
| UF R | 1.56 | 0.49 | 0.153 | 0.256 |
| SLF-temp L | 4.41 | 3.12 | 0.142 | 0.256 |
| SLF-temp R | -3.73 | -2.15 | 0.065 | 0.163 |

Abbreviations: ATR, anterior thalamic radiation; CST, corticospinal tract; CG, cingulum (cingulate gyrus); CH, cingulum (hippocampus); Fmaj, forceps major; Fmin, forceps minor; IFOF, inferior fronto-occipital fasciculus; ILF, inferior longitudinal fasciculus; SLF, superior longitudinal fasciculus; UF, uncinate fasciculus; L, left; R, right.

**Supplementary Table 15. Case-control differences in the global gradient metrics** **under stricter motion control (mean FD < 0.2 mm)**

| Metric | MDD (n=30),  mean (SD) | HC (n=33),  mean (SD) | *t* | Cohen’s *d* | *P* | FDR *q* |
| --- | --- | --- | --- | --- | --- | --- |
| G1 explained ratio | 0.093 (0.037) | 0.114 (0.047) | -2.01 | -0.51 | 0.0491 | 0.0491 |
| G1 range | 0.139 (0.033) | 0.156 (0.027) | -2.47 | -0.62 | 0.0166 | 0.0403 |
| G1 variance | 0.031 (0.011) | 0.036 (0.009) | -2.27 | -0.57 | 0.0269 | 0.0403 |
| G2 explained ratio | 0.072 (0.037) | 0.080 (0.043) | -0.79 | -0.20 | 0.4336 | 0.4336 |
| G2 range | 0.094 (0.034) | 0.105 (0.026) | -1.42 | -0.36 | 0.1618 | 0.3947 |
| G2 variance | 0.015 (0.009) | 0.018 (0.008) | -1.13 | -0.29 | 0.2631 | 0.3947 |
| G3 explained ratio | 0.088 (0.041) | 0.100 (0.038) | -1.22 | -0.31 | 0.2291 | 0.2291 |
| G3 range | 0.147 (0.030) | 0.174 (0.031) | -3.75 | -0.95 | 0.0004 | 0.0006 |
| G3 variance | 0.032 (0.010) | 0.041 (0.009) | -4.12 | -1.04 | 0.0001 | 0.0004 |

Abbreviations: FD, framewise displacement; MDD, major depressive disorder; HC, healthy controls; SD, standard deviation; G1, Gradient 1; G2, Gradient 2; G3, Gradient 3.

**Supplementary Table 16. Case-control differences in the gradient scores of the tracts in G2** **under stricter motion control (mean FD < 0.2 mm)**

| Tracts | *t* | Cohen’s *d* | *P* | FDR *q* |
| --- | --- | --- | --- | --- |
| ATR L | -2.96 | -0.25 | 0.004 | 0.015 |
| ATR R | -2.91 | -0.27 | 0.004 | 0.015 |
| CST L | 3.08 | 0.32 | 0.003 | 0.014 |
| CST R | -1.63 | -0.19 | 0.108 | 0.234 |
| CG L | 0.43 | 0.08 | 0.674 | 0.842 |
| CG R | 1.12 | 0.28 | 0.281 | 0.401 |
| CH L | 0.05 | 0.02 | 0.964 | 0.983 |
| CH R | -2.69 | -0.78 | 0.021 | 0.052 |
| Fmaj | -1.19 | -0.12 | 0.236 | 0.363 |
| Fmin | -6.07 | -0.36 | <1.0×10^-16^ | <1.0×10^-16^ |
| IFOF L | 0.21 | 0.02 | 0.838 | 0.983 |
| IFOF R | 0.02 | 0.00 | 0.983 | 0.983 |
| ILF L | -2.43 | -0.25 | 0.017 | 0.048 |
| ILF R | -5.04 | -0.67 | <1.0×10^-16^ | <1.0×10^-16^ |
| SLF L | 13.98 | 1.14 | <1.0×10^-16^ | <1.0×10^-16^ |
| SLF R | -0.54 | -0.05 | 0.593 | 0.791 |
| UF L | -1.64 | -0.36 | 0.117 | 0.234 |
| UF R | -1.57 | -0.50 | 0.151 | 0.275 |
| SLF-temp L | 0.10 | 0.07 | 0.934 | 0.983 |
| SLF-temp R | -1.93 | -1.11 | 0.194 | 0.323 |

Abbreviations: FD, framewise displacement; ATR, anterior thalamic radiation; CST, corticospinal tract; CG, cingulum (cingulate gyrus); CH, cingulum (hippocampus); Fmaj, forceps major; Fmin, forceps minor; IFOF, inferior fronto-occipital fasciculus; ILF, inferior longitudinal fasciculus; SLF, superior longitudinal fasciculus; UF, uncinate fasciculus; L, left; R, right.

**Supplementary Table 17. Case-control differences in the gradient scores of the tracts in G3 under stricter motion control (mean FD < 0.2 mm)**

| Tracts | *t* | Cohen’s *d* | *P* | FDR *q* |
| --- | --- | --- | --- | --- |
| ATR L | -4.23 | -0.36 | <1.0×10^-16^ | <1.0×10^-16^ |
| ATR R | -3.70 | -0.35 | <1.0×10^-16^ | 0.001 |
| CST L | -3.87 | -0.40 | <1.0×10^-16^ | <1.0×10^-16^ |
| CST R | -2.50 | -0.29 | 0.014 | 0.019 |
| CG L | 6.22 | 1.15 | <1.0×10^-16^ | <1.0×10^-16^ |
| CG R | 3.32 | 0.83 | 0.005 | 0.007 |
| CH L | 8.57 | 3.03 | <1.0×10^-16^ | <1.0×10^-16^ |
| CH R | 5.53 | 1.60 | <1.0×10^-16^ | <1.0×10^-16^ |
| Fmaj | -16.25 | -1.63 | <1.0×10^-16^ | <1.0×10^-16^ |
| Fmin | 28.67 | 1.68 | <1.0×10^-16^ | <1.0×10^-16^ |
| IFOF L | -2.19 | -0.23 | 0.031 | 0.039 |
| IFOF R | -2.53 | -0.24 | 0.013 | 0.018 |
| ILF L | -0.98 | -0.10 | 0.331 | 0.348 |
| ILF R | -3.33 | -0.44 | 0.002 | 0.003 |
| SLF L | -5.96 | -0.48 | <1.0×10^-16^ | <1.0×10^-16^ |
| SLF R | -8.47 | -0.75 | <1.0×10^-16^ | <1.0×10^-16^ |
| UF L | 6.16 | 1.34 | <1.0×10^-16^ | <1.0×10^-16^ |
| UF R | 2.33 | 0.74 | 0.045 | 0.053 |
| SLF-temp L | -8.71 | -6.16 | 0.073 | 0.081 |
| SLF-temp R | -0.86 | -0.49 | 0.482 | 0.482 |

Abbreviations: FD, framewise displacement; ATR, anterior thalamic radiation; CST, corticospinal tract; CG, cingulum (cingulate gyrus); CH, cingulum (hippocampus); Fmaj, forceps major; Fmin, forceps minor; IFOF, inferior fronto-occipital fasciculus; ILF, inferior longitudinal fasciculus; SLF, superior longitudinal fasciculus; UF, uncinate fasciculus; L, left; R, right.

**Supplementary Table 18. Case-control differences in the global gradient metrics in HKH**

| Metric | MDD (N=29),  mean (SD) | HC (N=27),  mean (SD) | *t* | Cohen’s *d* | *P* | FDR *q* |
| --- | --- | --- | --- | --- | --- | --- |
| G1 explained ratio | 0.125 (0.042) | 0.138 (0.040) | -1.21 | -0.32 | 0.2321 | 0.2321 |
| G1 range | 0.173 (0.039) | 0.187 (0.034) | -1.45 | -0.39 | 0.1538 | 0.2308 |
| G1 variance | 0.046 (0.039) | 0.053 (0.016) | -1.64 | -0.44 | 0.1065 | 0.2308 |
| G2 explained ratio | 0.130 (0.030) | 0.128 (0.035) | 0.19 | 0.05 | 0.8464 | 0.8464 |
| G2 range | 0.135 (0.024) | 0.141 (0.028) | -0.89 | -0.24 | 0.3778 | 0.6667 |
| G2 variance | 0.032 (0.008) | 0.034 (0.010) | -0.77 | -0.21 | 0.4445 | 0.6667 |
| G3 explained ratio | 0.012 (0.024) | 0.013 (0.028) | -0.20 | -0.05 | 0.8440 | 0.8440 |
| G3 range | 0.119 (0.019) | 0.125 (0.023) | -1.12 | -0.30 | 0.2673 | 0.5438 |
| G3 variance | 0.023 (0.005) | 0.024 (0.007) | -0.92 | -0.25 | 0.3625 | 0.5438 |

Abbreviations: MDD, major depressive disorder; HC, healthy controls; SD, standard deviation; G1, Gradient 1; G2, Gradient 2; G3, Gradient 3.
